# GmSop20 Functions as a Key Coordinator of the Oil‐To‐Protein Ratio in Soybean Seeds

**DOI:** 10.1002/advs.202505181

**Published:** 2025-07-18

**Authors:** Haowei Zheng, Xinkang Feng, Longlong Wang, Wentao Shao, Shiyu Guo, Duo Zhao, Jiajia Li, Long Yan, Long Miao, Bincheng Sun, Huihui Gao, Hongmei Qiu, Yu Hu, Linlin Kong, Robert M. Stupar, Ying‐hui Li, Li‐juan Qiu, Xiaobo Wang

**Affiliations:** ^1^ School of Agronomy Anhui Agricultural University Hefei 230036 China; ^2^ State Key Laboratory of Crop Gene Resources and Breeding/The National Key Facility for Crop Gene Resources and Genetic Improvement (NFCRI)/Key Laboratory of Grain Crop Genetic Resources Evaluation and Utilization (MARA) Institute of Crop Science Chinese Academy of Agricultural Sciences Beijing 100081 China; ^3^ Hebei Key Laboratory of Crop Genetics and Breeding National Soybean Improvement Center Shijiazhuang Sub‐Center Huang‐Huai‐Hai Key Laboratory of Biology and Genetic Improvement of Soybean Ministry of Agriculture and Rural Affairs Institute of Cereal and Oil Crops Hebei Academy of Agricultural and Forestry Sciences Shijiazhuang 050035 China; ^4^ Hulunbuir Institute of Agriculture and Animal Husbandry Hulunbuir 021000 China; ^5^ Soybean Research Institute Jilin Academy of Agricultural Sciences Changchun 130000 China; ^6^ Department of Agronomy and Plant Genetics University of Minnesota Saint Paul 55108 USA

**Keywords:** GWAS, natural variation, seed oil‐to‐protein ratio, soybean, TWAS

## Abstract

The seed oil‐to‐protein ratio has increased remarkably during soybean domestication; however, the principal genetic determinants governing this critical agronomic trait remain elusive. Integrated genome‐wide and transcriptome‐wide association studies (GWAS/TWAS) are conducted and identified *GmSop20* on chromosome 20 as a pivotal regulator of soybean seed oil and its protein content. Genetic diversity analysis reveals a domestication‐selected allele, *GmSop20^C^*, which has undergone intense artificial selection and is dominant in cultivars across northern China and the USA. This allele drives a substantial increase in the oil‐to‐protein ratio from 0.35 in ancestral lines to 0.47 in cultivars. Functional validation reveals that the knockout of *GmSop20* significantly reduces the ratio to 0.19, while its overexpression increases it to 0.64. Notably, while mutant lines exhibit a modest increase in total oil and protein content, overexpression maintains stable compositional levels. Mechanistically, GmSop20 directly activates *GmSWEET10a* expression, synergizing two artificially selected loci within a unified regulatory network to amplify sugar allocation from the seed coat to the embryo, thereby enhancing oil accumulation. The findings establish GmSop20 as a master regulator of seed composition and provide insights for custom‐designing soybean nutritional profiles, enabling the precise regulation of the oil‐to‐protein ratio through targeted manipulation of this key genetic module.

## Graphical abstract

This study investigates the seed oil‐to‐protein ratio as a comprehensive quality metric and identifies GmSop20 as a crucial regulatory gene through forward genetics methodologies. By integrating GmSop20 with superior haplotypes and using gene editing/overexpression, new strategies are provided for precisely tuning the seed oil‐to‐protein ratio, thereby contributing to the customized design of seed nutrient composition for future breeding.

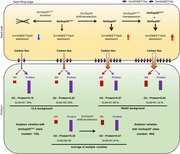

## 1 Introduction

Soybean (*Glycine max* [L.] Merr.) is one of the most important crops grown worldwide, accounting for 69% and 28% of the protein meal and vegetable oil consumed, respectively (http://soystats.com). Meeting the increasing demands of the growing population and rising standards of living will require a projected doubling of global soybean production by 2050.^[^ ^1^, ^2^, ^3^ ^]^ Therefore, the genetic improvement of high‐yielding and high‐quality soybeans is a significant breeding goal for human health maintenance and sustainable social development.

Cultivated soybean (*G. max*) has originated from domesticated wild soybean (*G. soja* Sieb. & Zucc.) in China for over 5000 years. Seed oil and protein content changed significantly during domestication and genetic improvement during the artificial selection process. The oil content is typically higher whereas the protein content is lower in cultivated soybeans than in wild soybeans. However, these values vary widely among soybean varieties.^[^ ^4^ ^]^ Seed composition traits are typically correlated, and seed protein content is often negatively correlated with seed oil content.^[^ ^5^, ^6^ ^]^ This inverse relationship poses a significant challenge for soybean breeding in terms of improving the overall economic value of soybeans. A better understanding of the molecular mechanisms that regulate the oil and protein balance using wild and local germplasms rich in variation would benefit this goal.

Soybean seed oil and protein content are both quantitative traits governed by multiple genes. Over the past 30 years, several quantitative trait loci (QTLs) associated with seed oil and protein content in soybeans have been identified using linkage analysis and genome‐wide association studies (https://soybase.org/).^[^ ^7^ ^]^ Several genes related to seed oil and protein contents have been validated in soybeans. This includes *GmSWEET10a*, which encodes a member of the SWEET family of sugar transporters and was previously designated as *GmSWEET39*.^[^ ^8^, ^9^, ^10^ ^]^ *GmSWEET10a* and its paralog *GmSWEET10b* controls sugar allocation from the seed coat to the embryo, affecting seed oil and protein content in soybean.^[^ ^11^ ^]^ *POWR1* encodes a CCT (CONSTANS, CONSTANS‐like, TOC1) motif‐containing protein that has pleiotropic effects on seed oil and protein content.^[^ ^12^, ^13^ ^]^ *GmMFT* encodes a phosphatidylethanolamine‐binding protein that modulates the expression of *SWEETs* and *FAD2*, thereby affecting the seed oil and protein content.^[^ ^14^, ^15^ ^]^ *GmFA9* encodes a SEIPIN homologue, and *GmFA9* knockout reduces the fatty acid content of soybean seeds and increases seed storage protein content.^[^ ^16^ ^]^ Previous studies have focused on the independent regulation of seed oil and protein content. However, the regulatory mechanisms underlying key seed quality traits, particularly the combined oil and protein content and the oil‐to‐protein ratio, remain largely unexplored.

In this study, we identified an oil and protein co‐determining gene, *GmSop20*, using forward genetics approaches of genome‐wide association study (GWAS) and transcriptome‐wide association study (TWAS). *GmSop20* knockout reduced the seed oil‐to‐protein ratio, whereas *GmSop20* overexpression increased this ratio. The artificially selected allele *GmSop20^C^* enhanced the activation of *GmSWEET10a/b*, resulting in improved sugar transport from the seed coat to the embryo, thereby increasing the oil‐to‐protein ratio in soybean seeds. This study revealed the mechanism by which *GmSop20* regulates the oil‐to‐protein ratio and identified the alleles that can be used to improve seed quality and enable customized nutrition in soybeans.

## 2 Results

### 2.1 Identification of a Candidate Gene Governing Seed Oil and Protein Content by GWAS and TWAS

To identify the genetic loci governing the quality of soybean seeds, the oil and protein contents of a diverse germplasm panel consisting of 1228 previously genotyped cultivated soybeans^[^ ^17^ ^]^ were phenotyped for three consecutive years (2018, 2019, and 2020) in He Fei (HF) City (31.8° N, 117.2° E) in Anhui Province and Shi Jia Zhuang (SJZ) City (38.5° N, 115.2° E) in Hebei Province, China (Tables S1 and S2, Supporting Information). The oil and protein content of the phenotypic accessions were significantly correlated (*P<0.001*) between the two sites (Figure S1, Supporting Information). Furthermore, there was a highly significant negative correlation (*R* = −0.85, *P* = 1.56e‐221) between oil and protein content, which is consistent with previous studies (Figure S1, Supporting Information).^[^ ^18^ ^]^ The broad‐sense heritability of oil and protein content obtained from three years and two sites of phenotypic characterization was high (0.76 and 0.61, respectively), indicating that these two traits are largely genetically controlled in soybean. These phenotypic data provided a basis for identifying the genetic loci controlling the oil and protein content in soybean seeds (Table S3, Supporting Information).

**Figure 1:**
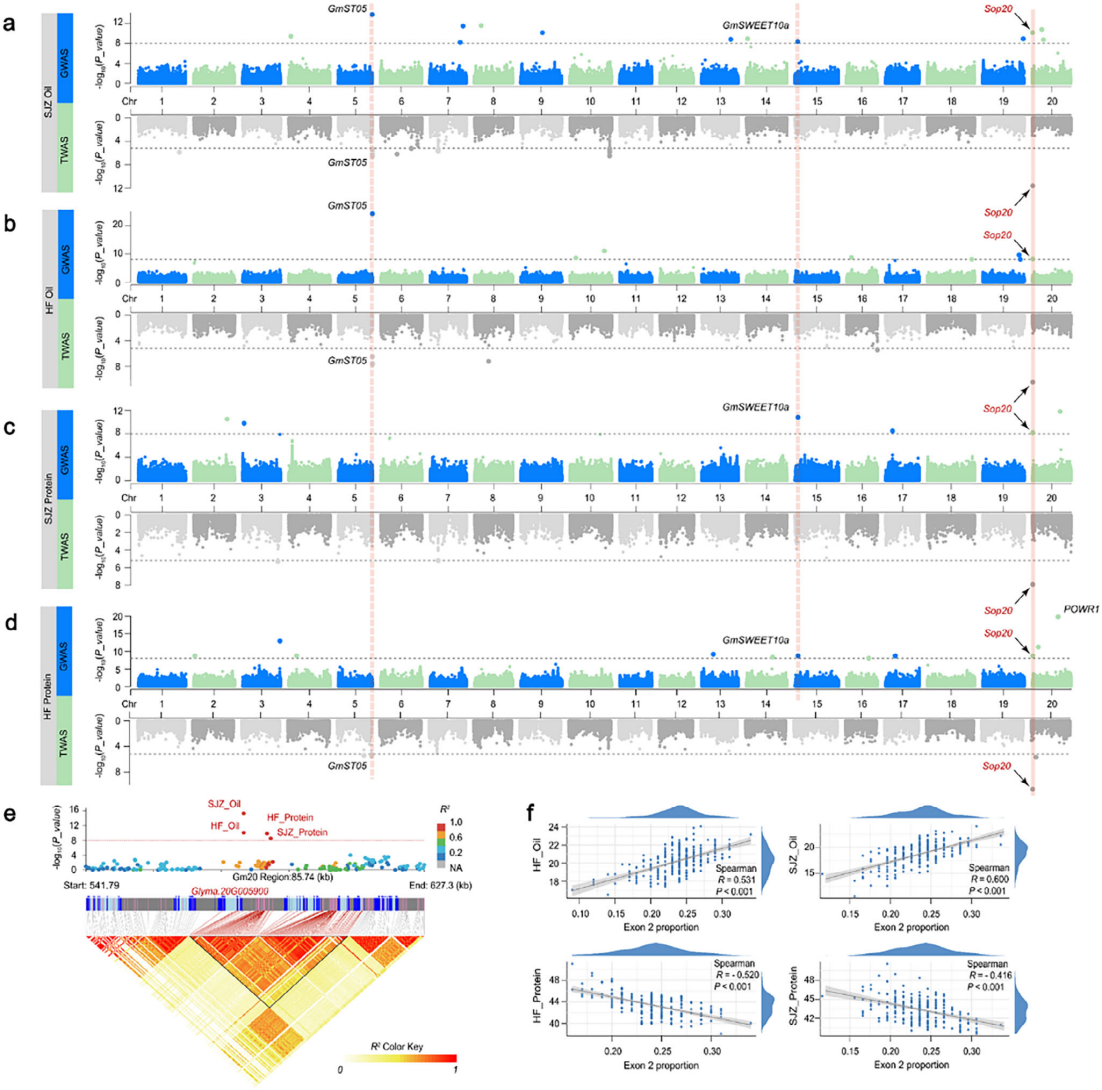
Identification of the major QTL, *GmSop20*, associated with soybean seed oil and protein. a–d) Manhattan plot of GWAS and TWAS for soybean oil in a) SJZ and HF (b), for soybean protein in c) SJZ and HF (d) (Tables S1 and S2, Supporting Information). Each spot represents a SNP in GWAS (top) or an exon in TWAS (bottom). The *P*‐values derived from association analyses conducted using FarmCPU (GWAS) and CMLM (TWAS) were log‐transformed, with the gray dashed line representing the Bonferroni correction threshold for multiple test adjustments. Previously identified genes are labeled in black and *GmSop20* in red. Blue and cyan spots alternate to distinguish different chromosomes. e) Linkage disequilibrium analysis of the *qSop20* locus, including a Manhattan plot of the 541–627 kb interval (correlations with PeakSNPs are indicated by the color of the spots), distribution of genes in the interval, and correlations between the two loci. f) Correlation analysis of the proportion of *Glyma.20G005900* exon 2 with seed protein and oil content in HF and SJZ. HF: He Fei city; SJZ: Shi Jia Zhuang city. Data were analyzed using Spearman's statistical method and the Shapiro–Wilk normality normal distribution test with 95% confidence intervals.

We performed a GWAS for oil and protein contents in this germplasm panel containing 4335175 SNPs using the fixed and random model circulating probability unification (FarmCPU) (**Figure** **1**a–d).^[^ ^19^ ^]^ The GWAS mapped 15 soybean protein QTLs (*qSP1‐qSP15*) and 19 soybean oil QTLs (*qSO1‐qSO19*) across both sites (Table S4, Supporting Information), including four QTL regions harboring the known functional genes *GmSWEET10a, POWR1, ST05, GmWRI1c*,^[^ ^11^, ^12^, ^15^, ^20^ ^]^ thus validating the effectiveness of our approach in identifying target loci or genes. Regarding protein content, two QTLs were stable at both sites, nine were specific to HF City, and four were specific to SJZ City (Table S4, Supporting Information). The site‐stable QTL *qSP6* (peak marker at position Chr15_3853402) included the known functional gene *GmSWEET10a* controlling oil and protein content. Another site‐stable QTL, *qSP9*, was located on chromosome 20 (peak marker at position Chr20_586312). For oil content, two site‐stable QTLs (*qSO1* and *qSO8*), six HF‐specific QTLs, and 11 SJZ‐specific QTLs were identified (Table S4, Supporting Information). *qSO1* (peak marker at position Chr5_41890181) included the known functional gene *ST05* controlling oil and protein content. Importantly, QTLs *qSO8* and *qSP9* had coincident QTL with peak markers at positions Chr20_581793 and Chr20_586312, respectively. Further linkage disequilibrium analysis revealed that *qSO8* and *qSP9* are located in the same genomic interval, with a length of 39.1 kb (Figure 1e). The genetic region surrounding *qSO8* has been identified as an oil‐associated QTL in previous studies,^[^ ^21^ ^]^ supporting the role of *qSO8* in regulating seed oil content. Therefore, this locus was named *Sop20* (soybean oil and protein QTL on Chr. 20). Within this genomic interval of *Sop*20, five protein‐coding genes were annotated based on the Wm82.a4.v1 reference genome (https://phytozome‐next.jgi.doe.gov) (Figure 1e).

**Figure 2:**
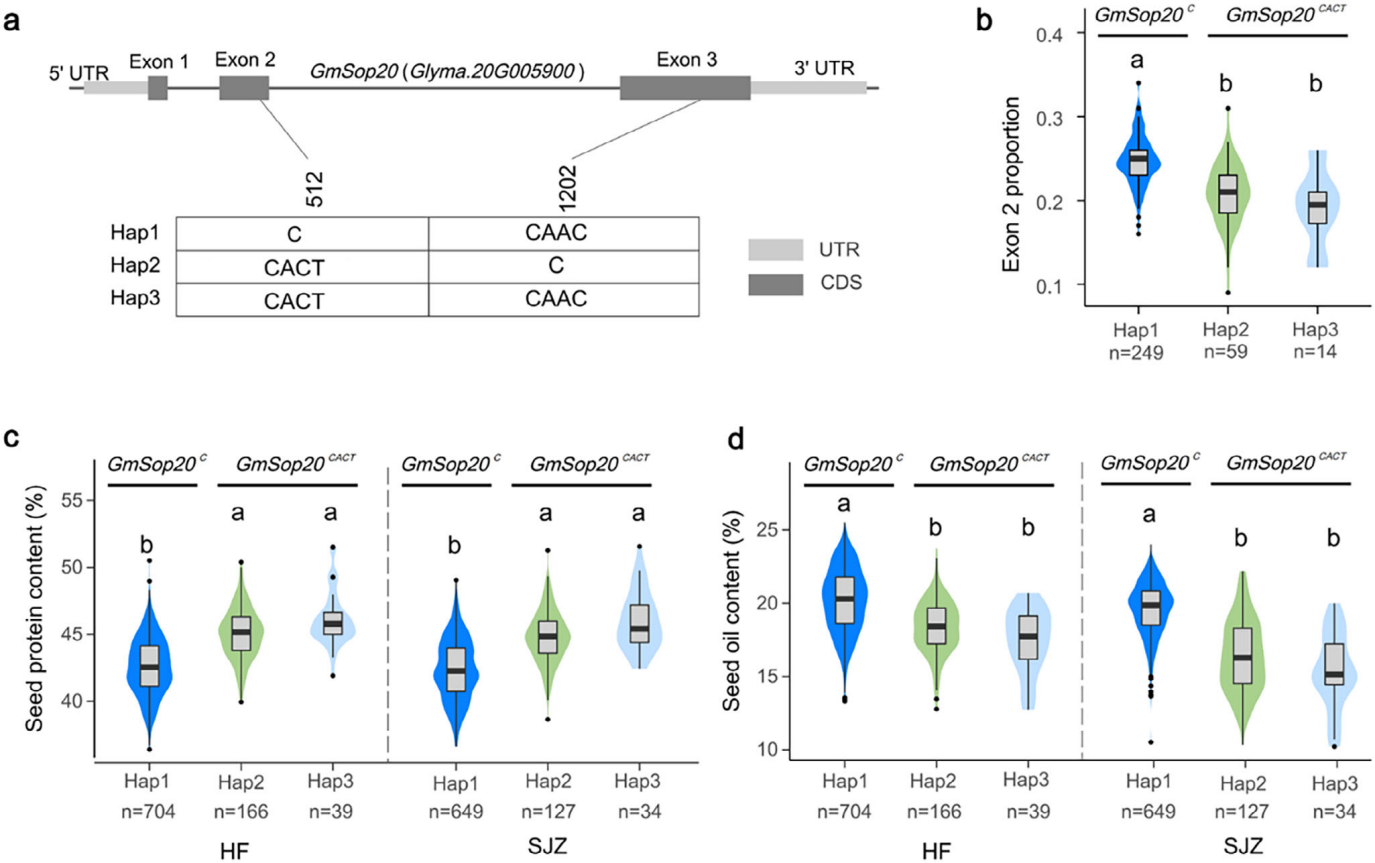
Allelic effect of *GmSop20* on seed oil and protein content in cultivated soybean. a) Haplotypes detected in the genomic region of *GmSop20*. b) Distribution of the second exon proportion for each haplotype. c,d) Distribution of seed traits for each haplotype, including seed protein content (c) and seed oil content (d). BLUP in HF city (left) and SJZ city (right). The BLUP values were calculated using the seed protein and oil content data of natural populations in two environments over three years (Methods‐GWAS and TWAS assays). The box plot shows the 25th to 75th percentile range, with a black line indicating the median. The whiskers extend to cover a range of 1.5 times the interquartile range, and black spots represent outliers. HF: He Fei city; SJZ: Shi Jia Zhuang city. Statistical significance was determined by one‐way ANOVA with Tukey's multiple‐comparison test.

To identify the candidate gene for *Sop20* using TWAS, seeds from a subset of the abovementioned diverse germplasm panel, comprising 199 landraces and 166 cultivars, were RNA‐sequenced at the R5.5 developmental stage of seed development (Table S1, Supporting Information). A total of 3.5 Tb of raw RNA‐seq data were generated, yielding an average of 26.8 million paired‐end reads per accession (Table S5, Supporting Information). These data produced informative exon proportion values for 154119 exons used to identify genes associated with oil and protein content by employing a compressed mixed linear model (CMLM).^[^ ^22^ ^]^ Exon proportion is a newly developed feature used to quantify both structural and alternative splicing variations in TWAS. It is calculated as the ratio of the exon read counts to the total read count of the corresponding gene.^[^ ^23^, ^24^ ^]^ Collectively, TWAS identified two soybean protein associated genes and four soybean oil genes in two locations, including two oil associated genes and one protein associated gene identified in both locations (Table S6, Supporting Information). One of the genes identified in both locations was the known functional gene *ST05* associated with oil content (Figure 1a–d).^[^ ^15^ ^]^ Another gene (*Glyma.20G005900*) identified at both locations was significantly associated with both oil and protein contents. *Glyma.20G005900* has three exons and two introns (**Figure** **2**a). Exon 2 of *Glyma.20G005900* was significantly positively correlated with seed oil content (Spearman correlation = 0.6, *P* < 0.001) and significantly negatively correlated with seed protein content (Spearman correlation = −0.52, *P* < 0.001) in the TWAS (Figure 1f; Figure S2a,b, Supporting Information). *Glyma.20G005900* is located within the *Sop20* GWAS locus and is thus designated *GmSop20*.

### 2.2 Genomic Variation of *GmSop20* is Correlated with Seed Oil and Protein Content

The sequence variation of *GmSop20* was analyzed in 1228 previously re‐sequenced cultivars.^[^ ^17^ ^]^ Two Indel variants located in the second (C‐CACT, resulting in an extra threonine) and third (CAAC‐C, resulting in the loss of aspartic acid) exons were detected in *GmSop20*, resulting in three haplotypes (Figure 2a). TWAS analysis of the 365 accessions revealed that the proportion of exon 2 was significantly lower in accessions carrying *GmSop20^Hap2/3^* than in those carrying *GmSop20^Hap1^* (Figure 2b; Figure S2c, Supporting Information). Meanwhile, accessions carrying *GmSop20^Hap1^* exhibited significantly higher oil content and lower protein content than those carrying *GmSop20^Hap2/3^* in both wild and cultivated soybeans (Figure 2c,d; Figure S3, Supporting Information). However, there were no significant differences in the protein and oil content or exon 2 proportion between those carrying *GmSop20^Hap2^* and *GmSop20^Hap3^*, which share the *GmSop20^CACT^* allele, in contrast to the *GmSop20^C^* allele in *GmSop20^Hap1^* (Figure 2a–d). This suggests that the (C to CACT) variation is responsible for the phenotypic transition. Molecular markers targeting the C/CACT locus have been developed to identify the genotype at this site (Figure S4, Supporting Information). In summary, *GmSop20^C^* is a high‐oil and low‐protein allele, and *GmSop20^CACT^* is a low‐oil and high‐protein allele.

### 2.3 *GmSop20* and its Paralog *GmSop07* Exhibit Increased Expression in the Seed Coat

**Figure 3:**
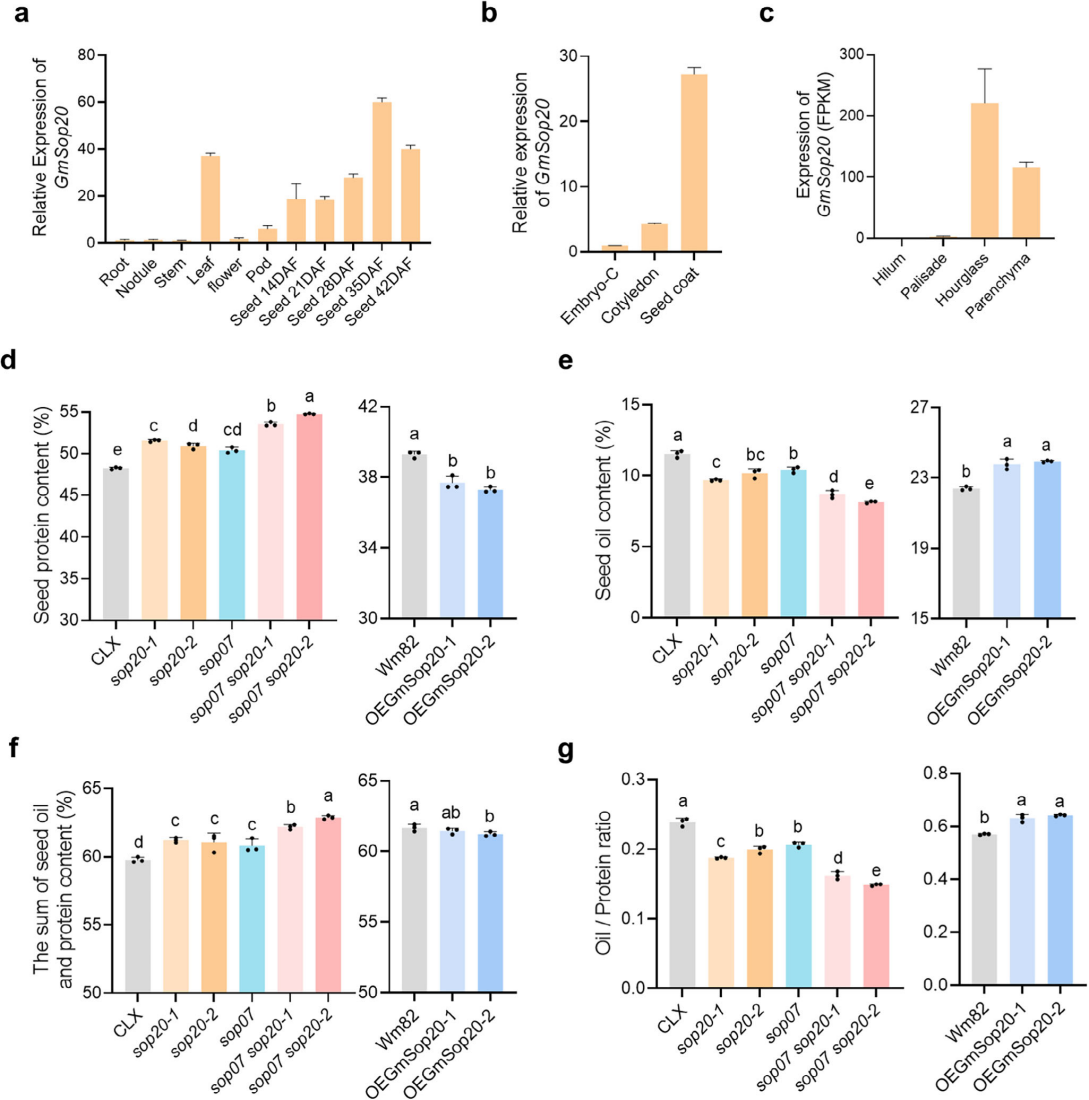
Effect of *GmSop20* and *GmSop07* on seed oil and protein content. a) Transcript abundance of *GmSop20* in different organs. b) Transcript abundance of *GmSop20* in embryos with the cotyledon removed (Embryo‐C), cotyledon, and seed coat. Developing seeds at 35 DAF were used for RNA extraction. c) Transcript abundance of *GmSop20* in Hilum, Palisade, Hourglass, and Parenchyma. Expression values were obtained from Gene Networks in Seed Development (http://seedgenenetwork.net/soybean). d–g) Protein content (d), oil content (e), the sum of oil and protein content (f), and the ratio of oil and protein (g) of mature seeds from wild type (CLX), *sop20*, *sop07*, double mutant *sop07 sop20* and wild type (Wm82), and *GmSop20^CACT^*‐overexpressing plants. FPKM, fragments per kilobase of exon per million mapped. Data shown in (a–g) represent mean ± SD (n = 3). Statistical significance was determined by one‐way ANOVA with Tukey's multiple‐comparison test.

We first analyzed the expression patterns of the *GmSop20* gene in different soybean tissues and seeds at different developmental stages using quantitative real‐time PCR (qPCR). *GmSop20* exhibited high expression levels in leaves and developing seeds, with the highest expression in seeds 35 days after flowering (DAF) (**Figure** **3**a). Next, we analyzed the expression of *GmSop20* in different parts of the developing seeds (35 DAF). The *GmSop20* gene showed higher expression in the seed coat than in the cotyledon and embryo (with the cotyledon removed), as determined by qPCR (Figure 3b). Gene expression profiles of different cell layers of the soybean seed coat have been previously analyzed in Gene Networks in Seed Development (http://seedgenenetwork.net/soybean). Analyses of this database showed that *GmSop20* is mainly expressed in the hourglass and parenchyma but not in the other cell layers of the seed coat (Figure 3c).

Phylogenetic analysis showed that *GmSop20* has a close paralog located on Chr. 07 (*Glyma.07G158200*), named *GmSop07*, with 89.2% similarity in the protein sequence (Figure S5, Supporting Information). Genetic diversity analysis revealed no non‐synonymous or indel mutations in the *GmSop07* exon. Only two indels (A to AT and AA to A) were detected in the *GmSop07* promoter region, which resulted in the formation of three haplotypes (Figure S6a, Supporting Information). However, there were no significant differences in seed quality traits among plants carrying *GmSop07* ^*Hap1*^, *GmSop07* ^*Hap2*^, or *GmSop07* ^*Hap3*^ (Figure S6b, Supporting Information). Further analysis revealed that *GmSop07* exhibited expression patterns similar to those of *GmSop20* (Figure S7a–c, Supporting Information). Although no significant correlation was observed between seed quality traits and *GmSop07* variants, *GmSop07* may also have functions similar to *GmSop20* because of their highly similar protein sequences and expression patterns.

### 2.4 Functional Validation of *GmSop20* and *GmSop07* for Seed Oil‐To‐Protein Ratio Regulation

TWAS analysis revealed that a low exon 2 proportion of *GmSop20* exhibits a strong correlation with high protein content in soybean seeds (Figure 1f). To further investigate the function of *GmSop20*, we performed CRISPR/Cas9‐mediated genome editing in a high‐protein (>46.57%; Table S2, Supporting Information) soybean variety, JiLinCaiLiXiang (CLX; carries the *GmSop20^CACT^* haplotype). We obtained two independent *sop20* mutant lines, *sop20‐1* and *sop20‐2*, which carried 1 bp and 7 bp frameshift deletions in *GmSop20* exon 2 (Figure S8a and Table S7, Supporting Information). Both mutations resulted in the formation of premature stop codons (Table S7, Supporting Information). Knockout of *GmSop20* significantly increased seed protein content from 48.25% to 51.24% (Figure 3d) and simultaneously reduced seed oil content from 11.53% to 9.92% (Figure 3e). The sum of the seed oil and protein content increased from 59.78% (48.25% protein + 11.53% oil) to 61.16% (51.24% protein + 9.92% oil; Figure 3f). The seed oil‐to‐protein ratio decreased from 0.24 to 0.19 (Figure 3g). Considering the similarity between *GmSop07* and *GmSop20*, we constructed *sop07* mutant and *sop20 sop07* double mutants (Figure S8b,c and Table S7, Supporting Information). Knockout of *GmSop07* showed significant effects on seed protein and oil traits, which were similar but weaker than those of the *sop20* knockout mutants (Figure 3d–g). Moreover, knockout of both *GmSop07* and *GmSop20* led to more violent effects, including significantly increased seed protein content from 48.25% to 54.13% (Figure 3d), and simultaneous reduction in seed oil content from 11.53% to 8.41% (Figure 3e). The sum of the seed oil and protein content increased from 59.78% (48.25% protein + 11.53% oil) to 62.54% (54.13% protein + 8.41% oil; Figure 3f). The seed oil‐to‐protein ratio decreased from 0.24 to 0.16 (Figure 3g).

To further confirm the function of *GmSop20*, we generated two overexpression lines and confirmed the presence of the selective bar gene by strip detection and qPCR analysis (Figure S9, Supporting Information). *GmSop20^CACT^* overexpression in the Williams 82 (Wm82; carries the *GmSop20^C^* haplotype) background reduced seed protein content from 39.29% to 37.49% (Figure 3d) and increased seed oil content from 22.39% to 23.85% (Figure 3e). The sum of the seed oil and protein contents was slightly reduced from 61.68%(39.29% protein + 22.39% oil) to 61.34%(37.49% protein + 23.85% oil; Figure 3f). Seed oil‐to‐protein ratio increased from 0.57 to 0.64 (Figure 3g). Collectively, the knockout of *GmSop20* and *GmSop07* reduced the seed oil‐to‐protein ratio, whereas the overexpression of *GmSop20* increased this ratio. The sum of seed oil and protein content slightly increased in *sop20* and *sop07* mutants, but was relatively stable in *GmSop20* overexpression lines. Therefore, our findings indicated that both *GmSop07* and *GmSop20* play essential roles in regulating the seed oil‐to‐protein ratio in soybeans.

### 2.5 GmSop20 Promotes *GmSWEET10a/b* Expression and Sugar Transport from the Seed Coat to the Embryo

**Figure 4:**
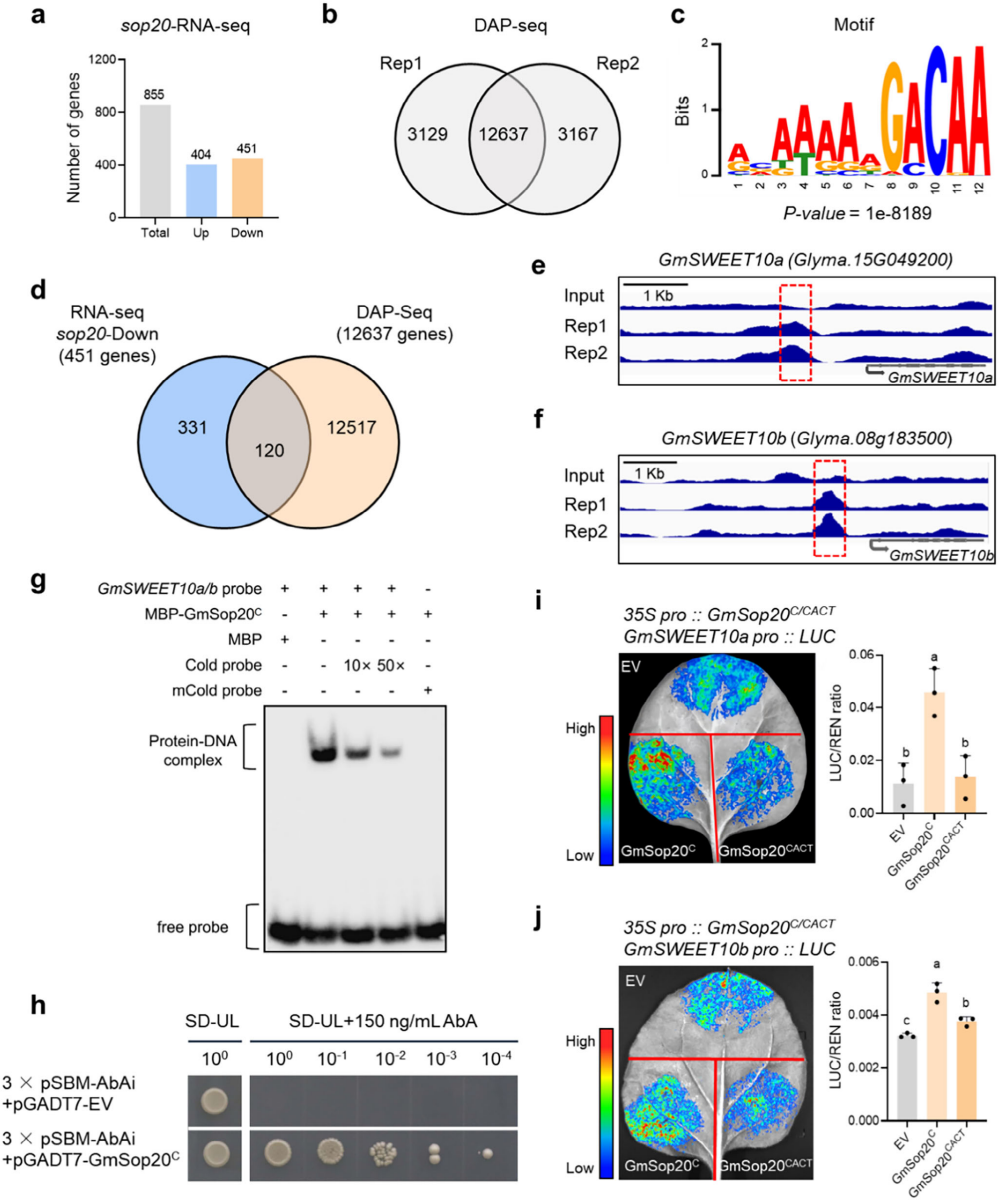
GmSop20 promotes the expression of *GmSWEET10a* and *GmSWEET10b*. a) Numbers of differentially expressed genes (DEGs) related to *GmSop20* knockout lines. Developing seeds at S5–S6 were used for RNA extraction. b) Overview of genes related to GmSop20 binding sites in the DAP‐seq of two biological replicates. c) A putative GmSop20‐binding motif identified in the promoter regions. The P‐value was calculated by MEME. d) Venn diagram of overlapping genes in two datasets: *sop20*‐DOWN and DAP‐seq. e,f) The localization of the GmSop20‐enriched binding peaks in the promoter regions of *GmSWEET10a* (e) and *GmSWEET10b* (f). g) an EMSA of MBP‐GmSop20^C^ protein binding to the *GmSWEET10a/b* promoter. MBP or MBP‐GmSop20^C^ fusion proteins were purified from *Escherichia coli*. Oligonucleotides (GCTCACACTATGAGACAAACGAGTA) were labeled with biotin. The probe sequence of *GmSWEET10a/b* was determined based on the location of the binding peaks in DAP‐seq results and is listed in Table S11 (Supporting Information). An excess of non‐labeled probe or mutated probe was used as a control. h) GmSop20^C^ bound to the *GmSWEET10a/b* promoter sequence (CTATGAGACAAA), as confirmed by Y1H. (SBM: GmSop20 binding motif in *GmSWEET10a/b* promoter; SD‐UL: SD/‐Ura/‐Leu) i,j) Luciferase‐activation assay in *N. benthamiana* leaves. The signal intensity shows the levels of different haplotypes of GmSop20 in activating firefly luciferase (LUC) reporter expression driven by the *GmSWEET10a* (i) and *GmSWEET10b* (j) promoters. EV, empty vector; The quantitative results are shown on the right. For (i, j), at least three independent experiments were performed. Similar results were observed. Data shown in (i, j) represent mean ± SD (n = 3). Statistical significance was determined by one‐way ANOVA with Tukey's multiple‐comparison test.

*GmSop20* encodes a C2H2‐type zinc‐finger transcription factor. RNA‐seq was performed to identify the targets of GmSop20. Total RNA was extracted from the R5‐R6 seeds of *GmSop20* knockout plants and compared with the parental control line. *GmSop20* knockout seeds exhibited differential expression of 855 genes (404 upregulated and 451 downregulated), with a fold change greater than 1.5 (**Figure** **4**a; Table S8, Supporting Information). Because *GmSop20* is a proposed transcriptional activator, the 451 downregulated genes in knockout plants (*sop20*‐DOWN) may represent its direct or indirect targets. Kyoto Encyclopedia of Genes and Genomes (KEGG) analysis revealed that these differentially expressed genes (DEGs) were largely involved in signal transduction, carbohydrate metabolism, lipid metabolism, amino acid metabolism, transport, and catabolism (Figure S10, Supporting Information).

Subsequently, DNA Affinity Purification Sequencing (DAP‐seq) was performed to capture GmSop20 regulatory targets at the whole‐genome scale. A total of 12637 target genes were identified within both biological replicates of the DAP‐seq experiment, with binding peaks located within the putative promoter regions of each gene (Figure 4b; Table S9, Supporting Information). Analysis of GmSop20 binding peaks by MEME identified an enriched motif containing a 12‐bp conserved sequence (Figure 4c). Furthermore, we identified 120 genes in both the *sop20*‐DOWN and DAP‐seq gene lists (Figure 4d; Table S10, Supporting Information). By searching the online expression database in Phytozome, we found that nine of the 120 genes showed enhanced expression in the seeds, similar to *GmSop20* (Figure S11a, Supporting Information). Among these, we first identified two previously reported genes, *GmSWEET10a* (*Glyma.15G049200*) and *GmSWEET10b* (*Glyma.08G183500*), which function together to control soybean seed oil and protein content (Figure S11b, Supporting Information).^[^ ^11^ ^]^ Additionally, *GmSWEET10a* and *GmSWEET10b* were mainly expressed in the parenchyma cells of the seed coat and showed highly overlapping expression patterns with *GmSop20* at different stages of seed development and in different parts of the seed (Figure S7d–i, Supporting Information). Significantly enriched GmSop20‐binding peaks were identified in the promoter regions of both *GmSWEET10a* and *GmSWEET10b* in both replicates of the DAP‐seq analysis (Figure 4e,f). Therefore, we conclude that GmSop20 may enhance the expression of both *GmSWEET10a* and *GmSWEET10b* during the seed‐filling stage.

To further confirm our hypothesis, electrophoretic mobility shift assays (EMSA) were performed to analyze the in vitro binding of GmSop20 to corresponding synthetic oligonucleotide probes (5′‐GCTCACACTATGAGACAAACGAGTA‐3′) containing the conserved sequences in the promoter regions of *GmSWEET10a/b*. GmSop20^C^ bound directly to the promoters of *GmSWEET10a* and *GmSWEET10b* (Figure 4g). We next performed yeast one‐hybrid (Y1H) experiments using a bait sequence with a total length of 36 bp containing three repeats of a 12‐bp DNA fragment (5′‐CTATGAGACAAA‐3′) shared by the *GmSWEET10a/b* promoters (Figure 4h). Next, we performed EMSA and Y1H experiments for GmSop20^CACT^ and found that GmSop20^CACT^ could also bind to the conserved motif in *GmSWEET10a/b* promoters and enhance their expression in vivo, similar to GmSop20^C^ (Figure S12, Supporting Information).

We also performed transactivation assays on *Nicotiana benthamiana* (*N. benthamiana*) leaves using both GmSop20^CACT^ and GmSop20^C^ as effectors. The promoter fragments of *GmSWEET10a* and *GmSWEET10b*, containing the binding motif of GmSop20, were used to drive the expression of a luciferase reporter gene. GmSop20^C^ significantly enhanced the expression of both *GmSWEET10a* and *GmSWEET10b*, whereas GmSop20^CACT^ showed weaker activation (Figure 4i,j). Moreover, the GmSop20‐Luciferase protein fusion experiment showed that the GmSop20^C^‐LUC fusion protein may be more stable than the GmSop20^CACT^‐LUC fusion protein (Figure S13a–c, Supporting Information). Subcellular localization experiments showed that GmSop20^C^‐GFP displayed stronger fluorescence intensity in the nucleus than GmSop20^CACT^‐GFP (Figure S13d,e, Supporting Information). Immunoblotting for GmSop20^C/CACT^‐GFP fusion proteins also indicated that GmSop20^C^ was more stable than GmSop20^CACT^ in *N. benthamiana* leaves (Figure S13f, Supporting Information). We then examined the mRNA levels of *GmSop20^C^* and *GmSop20^CACT^* in the corresponding tobacco leaves using qPCR and found that the mRNA level of *GmSop20^CACT^* was slightly higher than that of *GmSop20^C^*, supporting our inference of protein stability (Figure S13g, Supporting Information). Online prediction of the protein structure showed the GmSop20^C^ shows a more compact 3D configuration than GmSop20^CACT^ (Figure S13h, Supporting Information). Collectively, these results suggest that GmSop20 enhances the expression of *GmSWEET10a/b* and that *GmSop20^C^* is a powerful variant among the *GmSop20* alleles.

**Figure 5:**
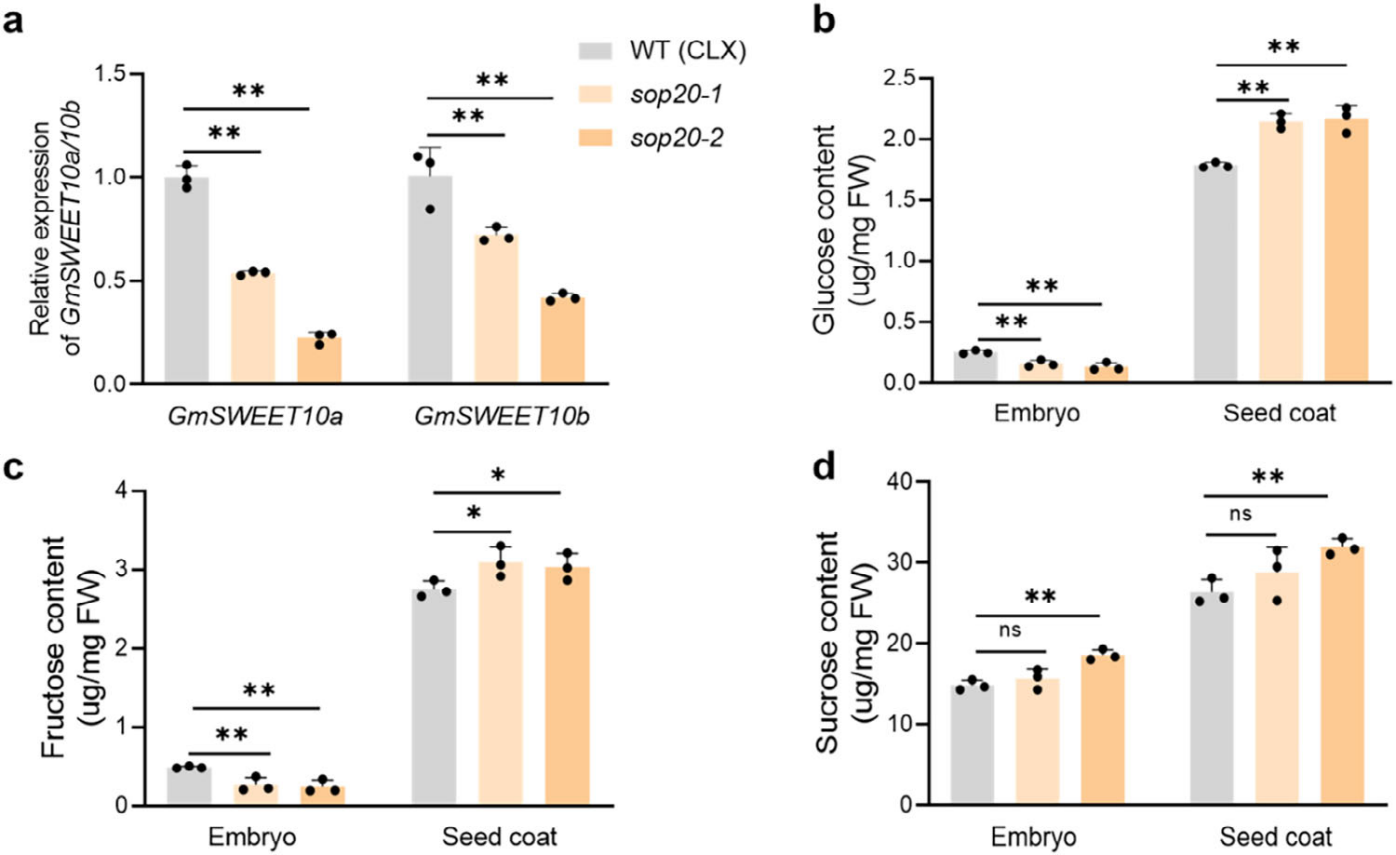
GmSop20 promotes sugar transport from the seed coat to the embryo. a) Relative expression levels of *GmSWEET10a/b* in wild type (CLX) and *sop20* mutant plants at 22 days after flowering (DAF). b–d) Sugar content in the embryos and seed coats of wild type (CLX) and *sop20* mutant plants at 22 DAF, including glucose (b), fructose (c), and sucrose (d). FW, Fresh weight. Data shown in (a–d) are mean ± SD (n = 3). *, *P *< 0.05; **, *P *< 0.01 of Student's *t*‐test. ns, not significant.

Previous studies have shown that the GmSWEET10a/b proteins mediate the transport of sucrose and hexose from the seed coat to the embryo, further stimulating oil accumulation in soybean seeds.^[^ ^11^ ^]^ qPCR revealed that *GmSWEET10a/b* transcription was downregulated in the seed (20‐22 DAF) of *sop20* knockout lines (**Figure** **5**a). The reduced oil‐to‐protein ratio in *sop20* mutants could be caused by the reduced availability of sugars in the embryo. To investigate this, sugar levels were measured in isolated seed coats and embryos at 20–22 DAF in CLX (wild type) and *sop20* mutants. Glucose and fructose levels were significantly lower in the embryos but significantly higher in the seed coats of *sop20* mutants compared to those of CLX (Figure 5b,c). In contrast, the sucrose content was slightly higher (but not consistently significant) in both the seed coat and embryo of the two *sop20* mutants compared to those of CLX (Figure 5d). These results indicated that the transport of glucose and fructose from the seed coat to the embryo was impaired in *sop20* mutants. These findings indicate that GmSop20 promotes sugar transport from the seed coat to the embryo by directly activating the *GmSWEET10a/b* genes. Accelerated carbon flux may further stimulate an increase in the oil‐to‐protein ratio in developing embryos.

### 2.6 *GmSop20* ^*C*^ was Artificially Selected in High‐Oil Soybean Breeding

**Figure 6:**
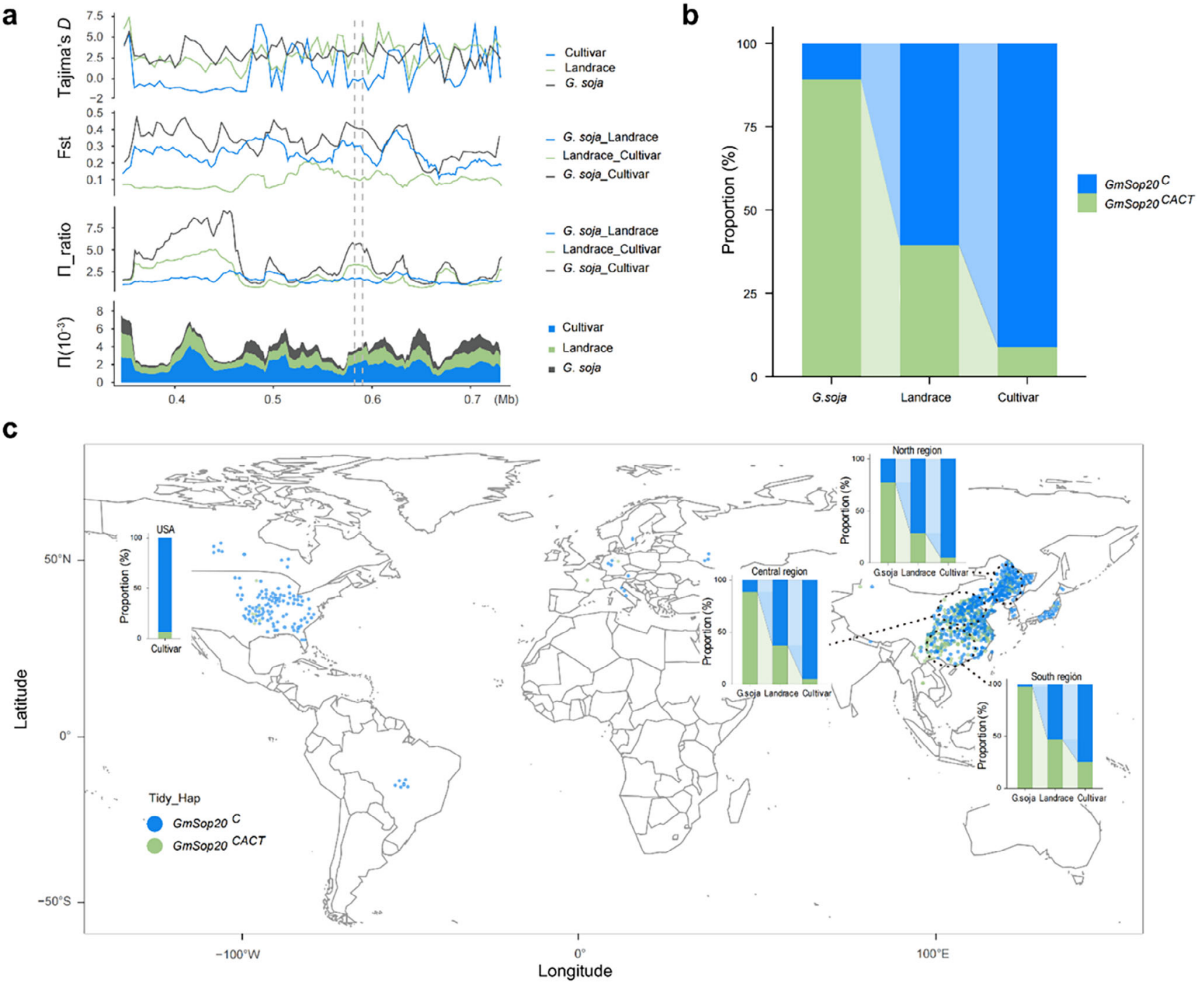
Evolution and geographical distribution of different haplotypes of *GmSop20*. a) Selective sweep analysis of the 0.345–0.735 Mb region on chromosome 20 containing *GmSop20*, comparing *G. soja* and *G. max* germplasm. Population genetic metrics shown are Tajima's *D, F*_st_, *π_*ratio, and *π*. Vertical black dashed lines indicate the physical location of *GmSop20*, and the color of the line indicates the species type. b) Haplotype frequency of *GmSop20* in *G. soja*, landrace, and cultivar lines. c) Haplotype frequency of *GmSop20* in different eco‐regions of China and the USA.

To gain initial insight into the molecular history of *GmSop20*, we performed a genome scan of the 390 kb (Chr20_345 000‐735 000) region adjacent to *GmSop20* using previously published data.^[^ ^17^ ^]^ *π* and *π*_ratio analyses showed that *G. soja* (1.62e‐3) exhibited higher diversity than landrace (1.36e‐3) and cultivar (0.45e‐3) soybeans. Tajima's *D* and *F*_st_ analyses indicated greater differentiation and distinct selection processes between wild and landrace soybeans, whereas less differentiation and a similar pattern of selection between landraces and cultivar soybeans (**Figure** **6**a). A negative Tajima's *D* and decreased genetic diversity were observed in only cultivar soybeans, suggesting that the *GmSop20* region underwent recent positive selection during soybean domestication (Figure 6a).

Given that oil and protein are important domestication traits, we investigated whether the *GmSop20* allele was selected during domestication and soybean improvement. We analyzed the frequency and geographical distribution of the two alleles in 129 *G. soja* and 1228 accessions cultivars that were previously re‐sequenced,^[^ ^17^ ^]^ all of which have genotypes and places of origin known worldwide (Table S2, Supporting Information). We found that the proportion of the low‐protein and high‐oil allele *GmSop20* ^*C*^ consistently increased from *G. soja* (10.85%) to landraces (60.47%), followed by cultivars (91.23%) (Figure 6b). This suggests that *GmSop20* ^*C*^ (low‐protein and high‐oil allele) has been subjected to intensive artificial selection during domestication and genetic improvement.

The continued increase in low‐protein and high‐oil alleles in cultivars compared to landraces prompted us to investigate their geographical distribution in the world, particularly in the USA and the Chinese regions (North, Central, and South regions). In China, the low‐protein and high‐oil alleles (*GmSop20^C^*) showed a gradually increasing trend from south to north, whereas the high‐protein and low‐oil alleles (*GmSop20^CACT^*) showed a decreasing trend (Figure 6c). Further analysis revealed that the low‐protein and high‐oil allele (*GmSop20^C^*) accessions were concentrated in the USA and Central and North regions of China in cultivars (Figure 6c). This suggests that *GmSop20^C^* is a key locus that has been widely selected for the high‐oil breeding of soybeans in both the USA and China.

### 2.7 A New *GmSop20‐GmSWEET10a/b* Module was Artificially Selected during Genetic Improvement of Seed Quality

**Figure 7:**
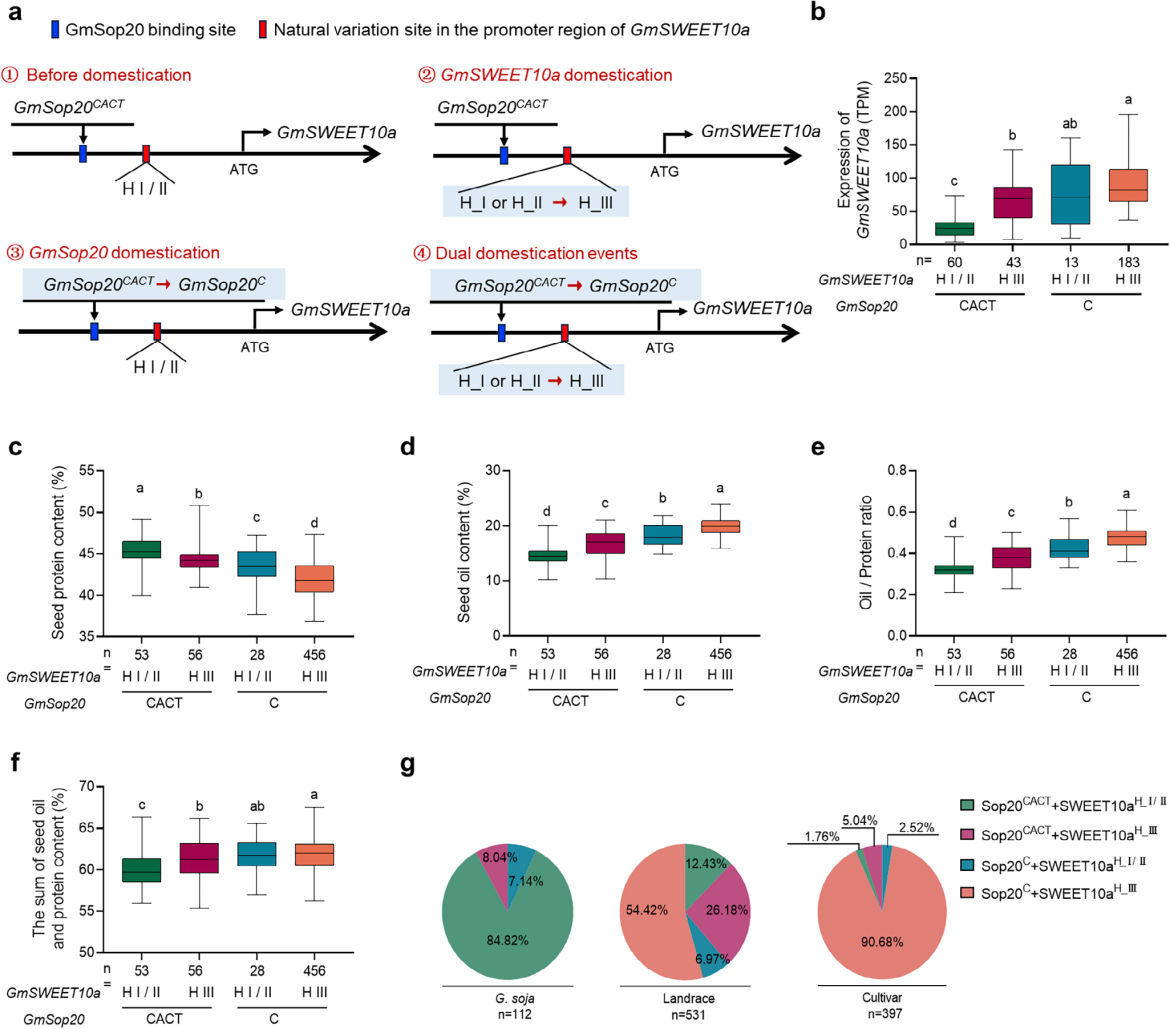
*GmSop20* mediated a new artificial selection event in regulating *GmSWEET10a* expression. a) Multi‐genic selection model for the *GmSWEET10a* expression regulation. The first selection event involves natural variation in the coding sequence of *GmSop20* (trans‐acting factor). The second selection event relates to variation in the promoter region of *GmSWEET10a* (cis‐acting element). Haplotypes carrying the respective selection alleles may exhibit enhanced expression of *GmSWEET10a* in the seed coat. b–f) Correlation analysis of the *GmSop20* and *GmSWEET10a* alleles with the expression level of *GmSWEET10a* (b), protein content (c), oil content (d), the ratio of oil and protein (e), and the sum of oil and protein content (f). g) Haplotype frequency of *GmSop20* and *GmSWEET10a* in *G. soja*, landrace, and cultivar lines. The box plot shows the 25th to 75th percentile range, with a black line indicating the median. The whiskers extend to cover a range of 1.5 times the interquartile range, and black spots represent outliers. Statistical significance was determined by one‐way ANOVA with Tukey's multiple‐comparison test.

Previous studies have reported that *GmSWEET10a* is a domesticated gene that contributes to fatty acid and protein content of soybean seeds.^[^ ^11^ ^]^ A 9‐base pair deletion in the promoter of *GmSWEET10a* was identified as a major variation site and was intensively selected during domestication.^[^ ^9^ ^]^ In the present study, we identified an upstream regulator of *GmSWEET10a*, GmSop20. Notably, the GmSop20 binding site was not located in the reported variation site (9‐base pair deletion) in the promoter region of *GmSWEET10a* (**Figure** **7**a). Considering *GmSop20* and *GmSWEET10a* are both domesticated or artificially selected genes, we propose that specific alleles of each gene were independently selected to regulate the transcription of *GmSWEET10a* (Figure 7a). For *GmSop20*, domestication involved a shift from *GmSop20^CACT^* to *GmSop20^C^*. For *GmSWEET10a*, the transition was from *GmSWEET10a^H_I/II^* to *GmSWEET10a^H_III^* (Figure 7a).^[^ ^11^ ^]^

Further analysis was performed to evaluate the correlation between the domestication alleles of *GmSop20* and *GmSWEET10a* and the resulting phenotypes (Table S2, Supporting Information). The transcription levels of *GmSWEET10a* were influenced by the genetic background of *GmSop20*, where *GmSop20^C^* produced higher expression than *GmSop20^CACT^* (Figure 7b). This transcriptional effect was greater than that observed for *GmSWEET10a^H_I/II^* and *GmSWEET10a^H_III^* (Figure 7b; Figure S14a, Supporting Information). The domesticated *GmSop20* allele resulted in a 2% reduction in seed protein content, which was greater than that of the *GmSWEET10a* domesticated allele (1.07%) (Figure 7c; Figure S14b, Supporting Information). The individual domesticated allele of *GmSop20* alone provided a 3.68% increase in seed oil content and a 0.1 increase in the seed oil‐to‐protein ratio compared with the *GmSop20^CACT^* allele, which was higher than the increase provided by the *GmSWEET10a* domesticated allele alone (2.15% and 0.06) (Figure 7d,e; Figure S14c,d, Supporting Information). Interestingly, although the sum of the seed oil and protein content significantly increased during domestication, the change was smaller than that of the oil‐to‐protein ratio (Figure 7e,f; Figure S14d,e, Supporting Information). Combining the domesticated *GmSop20^C^* and *GmSWEET10a^H_III^* alleles led to the strongest phenotype, producing the highest expression of *GmSWEET10a* (62.28 FPKM increase), oil content (5.3% increase), oil‐to‐protein ratio (0.16 increase), and total oil and protein contents (1.82% increase) (Figure S14a,c–e, Supporting Information). Haplotype frequency analysis showed that the proportion of *GmSop20^C^* +*GmSWEET10a^H_III^* was absent in *G. soja*, 54.42% in the landraces, and 90.68% in the cultivars (Figure 7g). These results indicate that *GmSop20* has a stronger function in regulating seed oil content and oil‐to‐protein ratio than *GmSWEET10a* during domestication, and the variants *GmSop20^C^* + *GmSWEET10a^H_III^* were co‐selected during high‐oil breeding.

**Figure 8:**
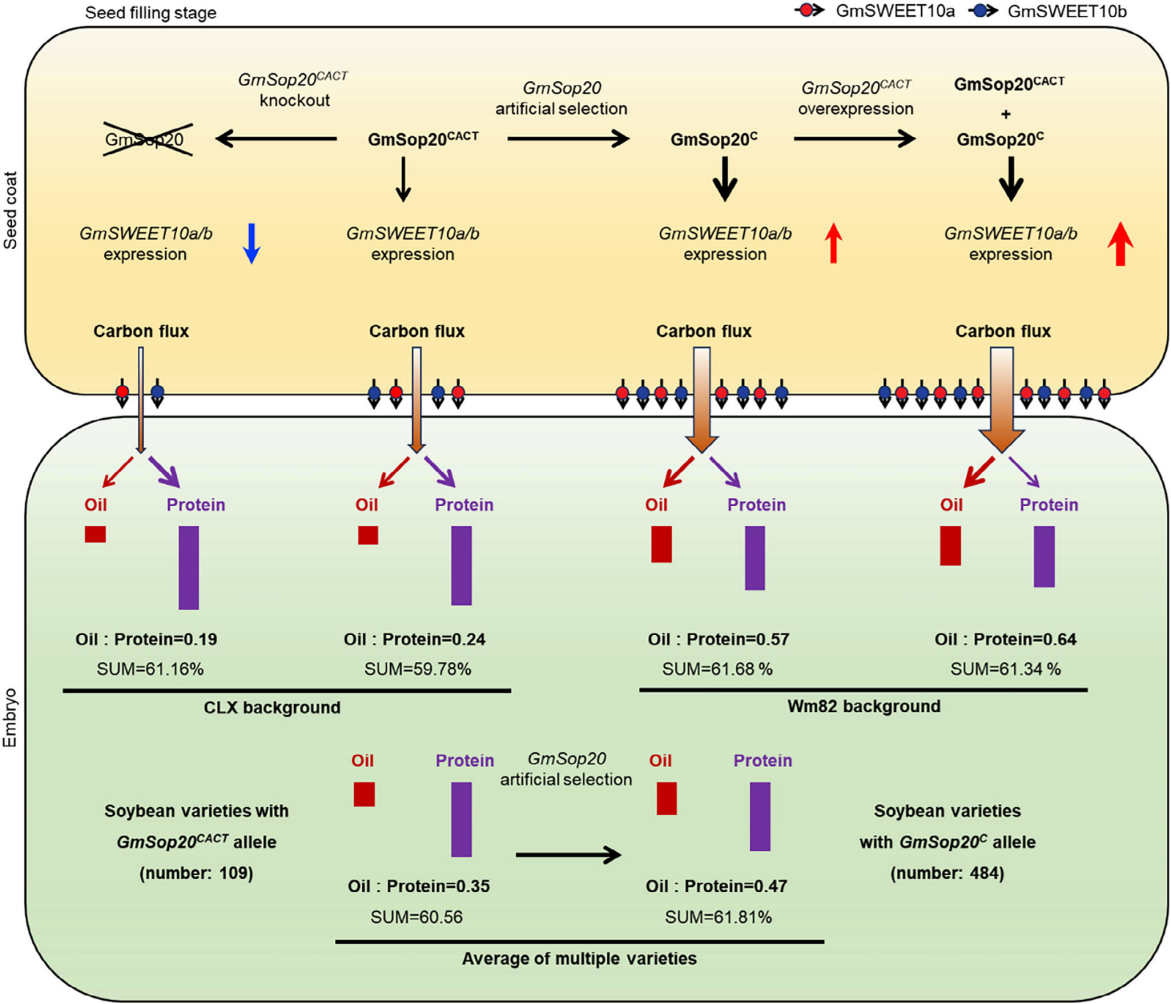
Schematic showing the mechanism by which GmSop20 regulates seed oil and protein content by enhancing the expression of *GmSWEET10a/b*. The deletion of ACT in the coding region results in increased stability of the GmSop20 protein, significantly enhancing the ability of GmSop20^C^ to activate *GmSWEET10a/b* compared to GmSop20^CACT^. This improvement facilitates a greater carbon flux from the seed coat to the embryo, leading to modifications in the oil and protein content due to higher carbohydrate levels. *GmSop20* knockout reduces, while *GmSop20* overexpression increases *GmSWEET10a/b* expression, thereby influencing the carbon flux from the seed coat to the embryo and affecting the oil and protein content. Brown arrows indicate the translocation of sugars from the seed coat to the embryo. Dark‐red arrows indicate the transformation of the carbon source into oil. Dark‐purple arrows indicate the transformation of the carbon source into protein. The red and blue arrows represent “increase” and “decrease”, respectively.

*GmSWEET10a/b* may accelerate carbon flux from the seed coat to the embryo.^[^ ^11^ ^]^ Therefore, we propose that artificial selection of *GmSop20* can improve carbon flux in the embryo and promote oil accumulation, which in turn increases the oil‐to‐protein ratio (0.35 to 0.47) (Figures 7e and **8**). *GmSop20^CACT^* knockout may reduce carbon flux in the embryo and retard oil accumulation, which, in turn, decreases the oil‐to‐protein ratio (0.24 to 0.19) (Figures 3g and 8). Overexpressing *GmSop20^CACT^* may improve carbon flux and increase the oil‐to‐protein ratio (0.57 to 0.64) (Figures 3g and 8). In all cases, the sum of the oil and protein content is relatively stable, ranging from 59.78% to 61.81% (Figures 3, 7, and 8). Overall, our findings elucidate a regulatory module involving the artificially selected gene *GmSop20*, which enhances the expression of *GmSWEET10a/b* for controlling the seed oil‐to‐protein ratio in soybean.

## 3 Discussion

The seed oil and protein content of cultivated soybeans are important agronomic traits frequently targeted by domestication and breeding efforts. These two traits are typically negatively correlated, complicating the interpretation of changes in one trait without considering the other. In this study, we first introduced a comprehensive indicator, the oil‐to‐protein ratio, that quantifies the relative proportion of oil and protein in soybean seeds. We used this metric to investigate the biological role of the *GmSop20* gene in regulating seed composition. Across diverse soybean genetic backgrounds, GmSop20 demonstrated a strong ability to regulate the seed oil‐to‐protein ratio, with values ranging from 0.19 to 0.64 (Figures 3, 7, and 8). Notably, the sum of oil and protein content remained relatively stable at ≈60% (Figures 3, 7, and 8). These results suggest that GmSop20 can modulate the oil‐to‐protein ratio without altering the total oil and protein content, emphasizing its potential utility in soybean breeding. Furthermore, this study highlights the importance of the oil‐to‐protein ratio as a crucial trait for future genetic improvement of soybean.

Although several genes, including *SWEET10a*, *POWR1, MFT*, *FA9*, *ST1*, *ZF351*, and *OLEO1*,^[^ ^11^, ^12^, ^14^, ^16^, ^25^, ^26^, ^27^ ^]^ have been reported to regulate the seed oil or protein contents in soybean, our understanding of the comprehensive regulatory mechanisms controlling these seed quality traits remains limited. In this study, we identified a novel gene, *GmSop20*, which plays a significant role in regulating the oil‐to‐protein ratio, a critical metric for evaluating seed quality, that has not been utilized in previous studies. Importantly, past studies have mainly focused on the function of individual genes without addressing the associations between these genes or the potential regulatory networks. This study demonstrated that the GmSop20 protein can directly target the promoter region of the previously identified oil and protein regulating gene *GmSWEET10a* (Figure 4g–j; Figure S12, Supporting Information). Interestingly, both *GmSop20* and *GmSWEET10a* were domesticated or artificially selected genes that cooperate within a regulatory module, representing a novel finding of two domestication genes or alleles functioning together (Figure 7a). Overall, our findings regarding the *GmSop20* and the *GmSop20‐GmSWEET10a* regulatory module provide new insights into the genetic mechanisms governing the oil‐to‐protein ratio in soybean seeds.

Domestication analysis revealed that a 3‐bp (ACT) deletion in the *GmSop20* coding region, located at the end of exon 2 and next to intron 2, underwent strong artificial selection. The 3‐bp (ACT) deletion showed a higher correlation with significantly lower coverage of reads in exons 1 and 2 than in exon 3 (Figure S2c, Supporting Information), reflecting a sharp contrast in exon‐specific expression. Such differential exon usage likely contributed to the significant TWAS signal observed in exon 2. Notably, the ACT deletion was positioned next to a predicted mRNA splicing site, suggesting a potential impact on splicing efficiency or the generation of alternative transcript isoforms. Interestingly, ACT deletion in the exon 2 coding sequence leads to a threonine deletion in the GmSop20 protein. Threonine is a potential phosphorylation site, suggesting the absence of ACT may result in the loss of a phosphorylation site in GmSop20^C^ (Figure S15, Supporting Information). In Arabidopsis, AtIDD8 is phosphorylated by SnRK1, which mediates flowering timing. AtIDD8 phosphorylation does not affect its subcellular localization and DNA‐binding properties but significantly reduces its transcriptional activity.^[^ ^28^ ^]^ In this study, we found that GmSop20^C^ with a threonine deletion showed higher transcriptional activation than GmSop20^CACT^, largely because of its enhanced protein stability (Figure S13a–g, Supporting Information). It is possible that GmSop20 lost this key residue during domestication, reducing the potential for protein phosphorylation, and thus enhancing protein stability and transcriptional activation. Therefore, the natural variation in ACT deletion in the *GmSop20* coding region may affect GmSop20 function at both the post‐transcriptional and post‐translational levels.

Wild soybeans have been successfully domesticated into a crop that is useful to humans, largely owing to continuing artificial selection. During the past two decades, reports of single‐gene domestication have become more frequent; however, there are a few reports of two or more domestication genes involved in the same signal transduction and/or metabolic pathway regulating or enhancing a specific trait in combination. In this study, we revealed GmSop20 is a regulator of the seed oil‐to‐protein ratio and works by directly activating *GmSWEET10a* expression (Figure 8), demonstrating two independent domestication events in a single regulatory module (Figure 7a). Interestingly, both genes in the domesticated form have greater effects than the interaction of either domestication gene with its undomesticated partner gene (Figure 7b–f). This indicates that these two domestication events synergistically control the expression of *GmSWEET10a* and together regulate the seed oil‐to‐protein ratio. In our survey of the soybean germplasm, over 90% of the cultivars carried a combination of these two genes in their domesticated forms, indicating the significance of the *GmSop20‐GmSWEET10a* module in modern breeding. Further elucidation of this regulatory mechanism may unlock new avenues for manipulating soybean seed oil‐to‐protein ratios using molecular and customized breeding approaches.

During seed maturation, the transport of carbon sources from the seed coat to the embryo is crucial for normal seed filling. A series of transport proteins, including those of the SWEET family, are involved in this process in different species.^[^ ^29^, ^30^, ^31^, ^32^ ^]^ Although these transport proteins have been identified in succession, the transcription factors that regulate their expression remain to be identified. To the best of our knowledge, this is the first study to identify a GmSop20 transcription factor that regulates the transport of carbon sources from the seed coat to the embryo by enhancing the expression of *GmSWEET10a/b* genes. Accordingly, both *sop20* and *sweet10a;10b* mutants exhibited the same trends of changes in seed oil and protein contents. As is reported, *GmSWEET10a/b* also significantly contribute to seed weight.^[^ ^11^ ^]^ However, both *GmSop20* and *GmSop07* exhibit weak regulatory effects on seed weight (Figure S16, Supporting Information). Additionally, the changes in sugar levels are much more pronounced in the *sweet10a;10b* double mutant than that in the *sop20* mutant (Figure 5),^[^ ^11^ ^]^ To explain these possible discrepancies, we propose that *GmSWEET10a/b* may not be solely regulated by GmSop20, because GmSop20 merely enhances the expression of *GmSWEET10a/b*. The regulatory factors may include GmSop07 and others that are yet to be identified. Interestingly, a recent study reported that Dt1 interacts with GmSWEET10a to control seed size without altering the oil and protein content of the soybean seeds.^[^ ^33^ ^]^ As a transcription factor, GmSop20 may regulate the expression of genes other than *GmSWEET10a/b*. Other candidate genes are shown in Figure S11b (Supporting Information). Overall, the relationship between GmSop20 and GmSWEET10a/b may involve other regulatory factors to form a signal transduction network that jointly regulates the diverse seed traits.

In summary, we identified *GmSop20* as the causal gene underlying the QTLs associated with seed oil and protein content in soybeans, based on locus identification using GWAS and TWAS. Our findings reveal that the *GmSop20* underwent artificial selection during soybean domestication and its effects on seed quality traits by enhancing the expression of *GmSWEET10a/b*. Furthermore, this study established the crucial role of the *GmSop20‐GmSWEET* *10* *a/b* module in regulating the seed oil‐to‐protein ratio in soybeans and highlights it as a representative case of multigenic selection for a quality trait during soybean domestication and improvement. The elucidation of this important epistatic relationship provides valuable insights for the strategic breeding and development of soybean cultivars with optimal seed composition.

## 4 Experimental Section

### Plant Materials and Growth Conditions

A total of 1228 plant materials were selected from 2214 previously sequenced lines from the soybean germplasm.^[^ ^17^ ^]^ These plant materials were planted in two different environments in HF, Anhui Province, China (31.8°N, 117.2°E), and SJZ, Hebei Province, China (38.5°N, 115.2°E), and phenotyped for three consecutive years in 2018, 2019, and 2020. Each cultivar was planted in two‐row plots of 1.8 × 0.8 m, with the seedlings in each row spaced 10 cm apart. The best linear unbiased predictive values (BLUP) for oil and protein were calculated separately for both plots using mixed linear models for GWAS analysis.

Under greenhouse conditions, *N. benthamiana* and soybean plants were grown in pots with nutritional soil under a 12‐h/12‐h (light/dark) photoperiod at 26/28 °C (night/day). For field experiments, transgenic lines in two genetic backgrounds—Wm82 (carries the *GmSop20* ^*C*^ haplotype), and CLX (carries the *GmSop20* ^*CACT*^ haplotype)—were planted every June and grown at the Anhui Agricultural University Farm (33.7° N, 117.1° E) in row plots (3‐m long, 0.4‐m row space, 30 plants per row for each accession).

### Measurement of Seed Traits

The protein and oil content of the 1228 soybean germplasm genotypes were determined using a near‐infrared grain quality analyzer (DA7200, Perten Instruments, Springfield, IL, USA). Different samples were analyzed in triplicate. The oil content of the transgenic plants was determined using solvent extraction techniques. Briefly, 2 g of the seed powder was extracted using isopropanol and hexane. Phase separation was performed by adding 15% (w/v) aqueous sodium sulfate. The upper and lower phases are separated from each other. The upper phase was collected. The lower aqueous phase, using 7/2 (v:v) hexane/isopropanol, and collected. The extracted oil was then evaporated to a constant weight under a nitrogen atmosphere. The oil content was calculated by dividing the weight of the extracted oil by the input seed weight. The protein content of the transgenic plants was determined using the Kjeldahl method. Briefly, the 100 mg seed powder was digested using 10 mL H_2_SO4 and a catalyst for 12 h, followed by stepwise heating at 160 °C for 15 min, 220 °C for 0.5 h, 350 °C for 0.5 h, and 450 °C for 2 h. Nitrogen content was determined using an automatic Kjeldahl apparatus (Kjeltec 8400; Foss, Hillerod, Denmark). The protein percentage was calculated by multiplying the nitrogen percentage by 6.25. The sum of the oil and protein contents was determined by summing the seed oil and protein contents. The oil‐to‐protein ratio was derived by dividing the seed oil content by the seed protein content. T5 homozygous transgenic knockout lines and T3 homozygous transgenic overexpression lines were used for the analysis.

### GWAS and TWAS

Genotype data were obtained using 8785134 previously published SNPs and imputed using Beagle. SNPs were filtered using minor allele frequency (MAF) >0.05, missing <20%, and Het <20% to obtain the final genotypes used for association analysis. Association analyses were performed using the FarmCPU model in the rMVP package for *R*.^[^ ^34^ ^]^ Population structure was controlled by the first three components of the SNP principal component analysis (PLINKv1.90).^[^ ^35^ ^]^ *P*‐value thresholds were based on the Bonferroni method and calculated using *P* = 0.05/SNP.

For RNA‐seq, we planted 365 soybean accessions (including 199 landraces and 166 cultivars) in a field in Sanya, Hainan, China (18.2°N, 109.9°E), with an average light duration of 11 h 20 min and a temperature of 22 °C (Table S5, Supporting Information). Fresh seed samples were collected from each accession at stage R5.5 of seed development, and all tissue samples were collected between 9:00 AM and 11:00 AM. Total RNA was extracted using the RNA Easy Fast Plant Tissue Kit (Tiangen Biotech, Beijing, China). Eukaryotic mRNA was enriched with oligo (dT) magnetic beads, and RNA‐seq libraries were constructed using the NEBNext Ultra RNA Library Prep Kit. Libraries were qualified using an Agilent 2100 Bioanalyzer and sequenced on an Illumina NovaSeq 6000 platform. Fastp^[^ ^36^ ^]^ was used for quality control and removal of low‐quality bases, and Hisat2^[^ ^37^ ^]^ aligned the paired‐end reads after quality control to the W82.a4.v1 reference genome^[^ ^38^ ^]^ using uniquely aligned reads for subsequent analyses. Reads for whole genes and coding exons were obtained using featureCounts.^[^ ^39^ ^]^

To capture variations in exon usage that reflect changes in expression, structural variation, and alternative splicing, exon proportions were used, calculated as the ratio of read mapping to a given exon relative to the total read mapping to the gene, based on Li's method.^[^ ^24^ ^]^ Exon proportions were calculated for genes with at least two exons, excluding exons with zero counts in > 95% of the accessions. These informative exon proportions were used for subsequent TWAS analyses using the CMLM method.^[^ ^22^ ^]^ The population structure was controlled by the first three principal components of the expression data. The resulting *P*‐value was adjusted at the α = 0.05 level using the Bonferroni correction.

### Genetic Diversity Analyses

SNP data from the previous studies were used to analyze genetic diversity in soybeans. SNPs with >10% missing data or a MAF 0.01 were excluded. Soybean germplasm was divided into three populations: *G. soja*, landraces, and cultivars. *F*_st_ values were calculated using VCFtools^[^ ^40^ ^]^ with a 5‐kb to 2‐kb sliding window to obtain genomic divergence coefficients between populations. Reduction in genetic diversity was estimated using nucleotide diversity (*π*) with a 5‐kb to 2‐kb sliding window. Tajima's *D* was calculated using high‐quality SNPs without applying an MAF filter to test whether DNA sequences followed a neutral evolutionary model.

### RNA Extraction and qPCR Expression Analyses

Total RNA was extracted using the RNA Easy Fast Plant Tissue Kit (TaKaRa, Otsu, Japan). First‐strand cDNA was synthesized using a HiScript III RT SuperMix kit (VAZYME, Nanjing, China), and qPCR analysis was performed using gene‐specific primers (Table S11, Supporting Information). qPCR analysis was performed using a CFX96 Real‐time System (Bio‐Rad, Hercules, CA, USA). Relative transcript levels were calculated using the 2^−ΔΔCt^ method^[^ ^41^ ^]^ by normalization to *GmACTIN11* expression^[^ ^42^ ^]^ in soybean. All qPCR analyses were performed in triplicate. The Wm82 cultivar was used for tissue‐specific expression assays. Plants were all planted under short‐day conditions, and tissues from root, nodule, stem, leaf, flower, pod, seed, seed coat, embryo‐c and cotyledon were collected for expression analysis of *GmSop20*, *GmSop07*, *GmSWEET10a* and *GmSWEET10b*.

### Plasmid Construction and Plant Transformation

To generate CRISPR/Cas9‐mediated mutations, a guide RNA (gRNA) target was designed using CRISPR‐GE (http://skl.scau.edu.cn/home/). The target sequence (5′‐CAAAACCTGTGGCACTAGAG‐3′) was designed to be in the conserved region of both *GmSop20* and *GmSop07* in the second exon. The target sequence was synthesized and cloned into pBlu/gRNA. The construct was digested with EcoRI, and the fragment containing the gRNA was transferred to the CRISPR/Cas9 binary vector.

To generate the *GmSop20* ^*CACT*^ overexpressing line, the coding sequence (CDS) of *GmSop20* ^*CACT*^ was amplified from cDNA derived from seed of CLX. The CDS was cloned into the 35S‐TNOS vector (pBWA(V)BS) with a BsaI site to generate a new construct *35S::GmSop20* ^*CACT*^. The primers used are listed in Table S11 (Supporting Information). The above constructs were introduced into Agrobacterium EHA105 for soybean transformation. Agrobacterium‐mediated transformation was performed as previously reported.^[^ ^43^ ^]^

### RNA‐Seq Profiling and Analysis

Total RNA was extracted from the seeds 28–32 days after fertilization. Seeds were collected from non‐transgenic and transgenic CLX lines (*sop20* mutants), with three biological replicates. Illumina HiSeq2500 was used to construct and sequence paired‐end libraries. The RNA‐seq reads were mapped to the soybean genome using Hisat2 after quality control.^[^ ^37^ ^]^ After sorting and indexing, fragments per kilobase of exon per million mapped reads (FPKM) were calculated using Cuffdiff to confirm DEGs with a fold change >1.5.

### DAP‐Seq Profiling and Analysis

Genomic DNA was extracted from leaves using a plant genomic DNA kit (Tiangen) with two biological replicates, and a Rapid DNA‐seq kit (BioRun Hubei Biotech, Wuhan, China) was used to construct the library. The DAP‐seq experiments were performed according to a published protocol.^[^ ^44^ ^]^ Briefly, the GmSop20‐HaloTag fusion protein was incubated with the constructed DNA library at 25 °C for 1 h, following which the GmSop20 fusion protein was immobilized using HaloTag beads and removal of unbound DNA fragments by washing. The GmSop20‐binding DNA fragments were recovered and subjected to PCR analysis. An Illumina NovaSeq 6000 instrument was used for sequencing, and reads were mapped to the* Glycine max* Wm82 genome v.4.0, using Bowtie2.^[^ ^45^ ^]^ For identifying GmSop20‐binding sites, MACS2 software was used to screen DAP‐seq peaks (fold enrichment > 2).^[^ ^46^ ^]^ Subsequently, Homer software^[^ ^47^ ^]^ was used to analyze the localization of DAP‐seq peaks within the 2 kb region upstream of the transcription start site. BEDtools^[^ ^48^ ^]^ was used to identify the GmSop20 targeted genes. The MEME‐ChIP suite 5^[^ ^49^ ^]^ was used to identify the motifs.

### Electrophoretic Mobility Shift Assay

The coding sequence of *GmSop20^C^* was amplified and inserted into the pMAL‐c5x‐MBP vector to generate recombinant MBP‐GmSop20^C^. The resulting construct was expressed in *Escherichia coli* strain BL21(DE3), and the recombinant protein was expressed and purified using Ni‐IDA resin according to the manufacturer's instructions. To prepare the probes, biotin‐labeled, competitive DNA fragments and mutated competitive DNA fragments were generated by annealing the corresponding oligonucleotides. The competitive and mutated probes (5–10 pm) and nonspecific competitor poly (dI‐dC) (0.1 µg µL^−1^) were mixed with the recombinant protein (250–500 ng) in a 5×EMSA buffer (100 mm HEPES, pH 7.4, 250 mm KCl, 10 mm MgCl_2_, 2.5 mm EDTA, 100 ng µL^−1^ of BSA, 1% NP‐40, 50% glycerol), and the mix was incubated at 25 °C for 10 min. Subsequently, biotin‐labeled probe was added, incubated at 25 °C for 20 min, and electrophoresed on 5% native polyacrylamide gels in 0.5×TBE. Fluorescence was detected using the Starion FLA‐5100 (Fujifilm). The related probes are listed in Table S11 (Supporting Information).

### Yeast‐One Hybrid

A sequence containing three tandem repeats of a 12‐bp DNA fragment (5′‐CTATGAGACAAA‐3′), which is shared by both the *GmSWEET10a/b* promoters, was amplified and inserted into the pAbAi vector to form the recombination plasmid Y1H‐GmSWEET10a/b. The Y1H‐GmSWEET10a/b plasmid was integrated into the genome of the yeast (*Saccharomyces cerevisiae*) strain Y1H Gold via homologous recombination to create a bait reporter strain. It is found that 150 ng mL^−1^ AbA inhibited the basal activation of Y1H Gold. The *GmSop20^C^* CDS fragment was amplified and inserted into the effector plasmid pGADT7, which was then co‐transformed into yeast strain Y1H‐GmSWEET10a/b. Transformants were cultivated on SD/‐Ura/‐Leu (including 150 ng mL^−1^ AbA) medium for 4–5 d. The empty pGADT7 vector was co‐transformed into yeast strain Y1H‐GmSWEET10a/b and used as a negative control.

### Transactivation Assays in *N. benthamiana*

For transactivation assays, recombinant plasmids were introduced into *Agrobacterium tumefaciens* GV3101. The effector constructs (EV, GmSop20^C,^ and GmSop20^CACT^) were paired with reporter constructs containing *GmSWEET10a* and *GmSWEET10b* promoters. Three combinatory constructs were simultaneously transformed into *N. benthamiana* leaves to measure promoter activation.^[^ ^50^ ^]^ The luminescence signal was captured using a Tanon 5200 electrochemiluminescent system after spraying the *N. benthamiana* leaves with 1 mm D‐luciferin potassium salt (VAZYME, Nanjing, China). The LUC/REN ratios were determined using a dual luciferase reporter gene assay kit (VAZYME, Nanjing, China). At least three independent leaves were examined, and each experiment was repeated thrice to confirm reproducible results.

### Subcellular Localization Assay

The CDS of *GmSop20^C^* and *GmSop20^CACT^* were amplified from soybean accessions (Wm82 and CLX). The CDS of *GmSop20^C^* and *GmSop20^CACT^* were inserted into pCAMBIA2300 in‐frame between the 35S promoter and the GFP protein. This recombinant plasmid was introduced into *Agrobacterium tumefaciens* GV3101 and infiltrated into *N. benthamiana* leaves for 48 h. Fluorescence signals (GmSop20^C^‐GFP and GmSop20^CACT^‐GFP) in *N. benthamiana* leaves were captured using a laser scanning confocal microscope (Zeiss LSM880; Carl Zeiss, Jena, Germany).

### Immunoblot Analysis

Fresh *N. benthamiana* leaves were collected, homogenized in liquid nitrogen, and homogenized in protein extraction buffer (1 mm PMSF, 10% [v/v] glycerol, 150 mm NaCl, 0.1% [v/v] Triton X‐100, 1× complete protease inhibitor cocktail [Roche], 1 mm EDTA, and 50 mm HEPES [pH 7.5]). The following antibodies were used: anti‐GFP (Roche, 11814460001, 1:1000 dilution) and horseradish peroxidase goat anti‐mouse (Abclonal, AS003, 1:5000 dilution). The wet transfer method was used for the PVDF membranes. Immunoblotting was performed using an enhanced chemiluminescence (ECL) system.

### Protein Structural and Phosphorylation Site Analysis

The 3D structures of GmSop20^C^ (GmSop20^Hap1^), GmSop20^CACT^ (GmSop20^Hap2/3^), and related proteins were predicted using the D‐I‐TASSER server with the default parameters (https://zhanggroup.org/D‐I‐TASSER/). Phosphorylation site prediction of GmSop20^C^ and GmSop20^CACT^ was performed using the web resource NetPhos 3.1 (https://services.healthtech.dtu.dk/services/NetPhos‐3.1/), which predicts serine, threonine, or tyrosine phosphorylation sites in eukaryotic proteins using ensembles of neural networks after inputting the amino acid sequence in FASTA format. The residue to be predicted was set to threonine. For each residue, only the best prediction was selected. Phosphorylation sites with a score higher than 0.5 were selected.

### Soluble Sugar Analyses

Developing seeds of *GmSop20* mutants and wild‐type (CLX) plants grown in a greenhouse were collected at 20–22 DAF. Seed coats and embryos (100 mg each) of non‐transgenic and transgenic CLX lines (*sop20* mutants) were placed separately in a 2 mL tube containing 700 µL 80% ethanol (v/v). The tubes were placed in a 50 °C water bath for 2 h, following which, 700 µL of water was added to the tube and the mixture was centrifuged at 12 000 rpm for 10 min. The upper phase was then transferred to a new tube. The samples were analyzed using HPAEC/PAD (Dionex ICS 5000; Thermo Scientific, Waltham, USA), as described by Jiang.^[^ ^51^ ^]^ The sugar types were identified according to the retention times of the standards, and the sample concentration was calculated using an external standard curve. Data were analyzed using three biological replicates.

### Statistical Analysis

Data were analyzed using GraphPad Prism 8.0. The Student's *t*‐test was utilized to analyze significant differences between two groups. Significant differences among the samples were determined using one‐way ANOVA with Tukey's multiple comparison test.

## Conflict of Interest

The authors declare no conflict of interest.

## Author Contributions

H.Z., X.F., and L.W. contributed equally to this work. X.W., Y‐H.L., and L‐J.Q. designed this study. H.Z., X.F., L.W., W.S., D.Z., Y.H., and L.K. performed the experiments. H.Z., X.F., S.G., and W.S. provided the relevant experimental materials. D.Z., J.L., L.M., L.Y., B.S., H.G., H.Q., and Y.H. assisted with the collection of phenotypic data. H.Z., X.F., L.W., Y‐H.L., and X.W. analyzed the data. Y‐H.L., L‐J.Q., L.W., H.Z., X.F., and R.M.S. wrote the manuscript.

## Supporting information



Supporting Information

Supporting Information

Supporting Information

Supporting Information

Supporting Information

Supporting Information

Supporting Information

Supporting Information

Supporting Information

Supporting Information

Supporting Information

Supporting Information

## Data Availability

The data that support the findings of this study are available in the supplementary material of this article.
