# Supplementary material for: GmSop20 Functions as a Key Coordinator of the Oil‐To‐Protein Ratio in Soybean Seeds
Source: Adv Sci (Weinh). 2025 Jul 18;12(38):e05181. doi: 10.1002/advs.202505181 (PMC12520551; doi:10.1002/advs.202505181)
Supplement: Supplementary file 1 — Supporting Information [file ADVS-12-e05181-s004.docx]

**Supplementary Figures**


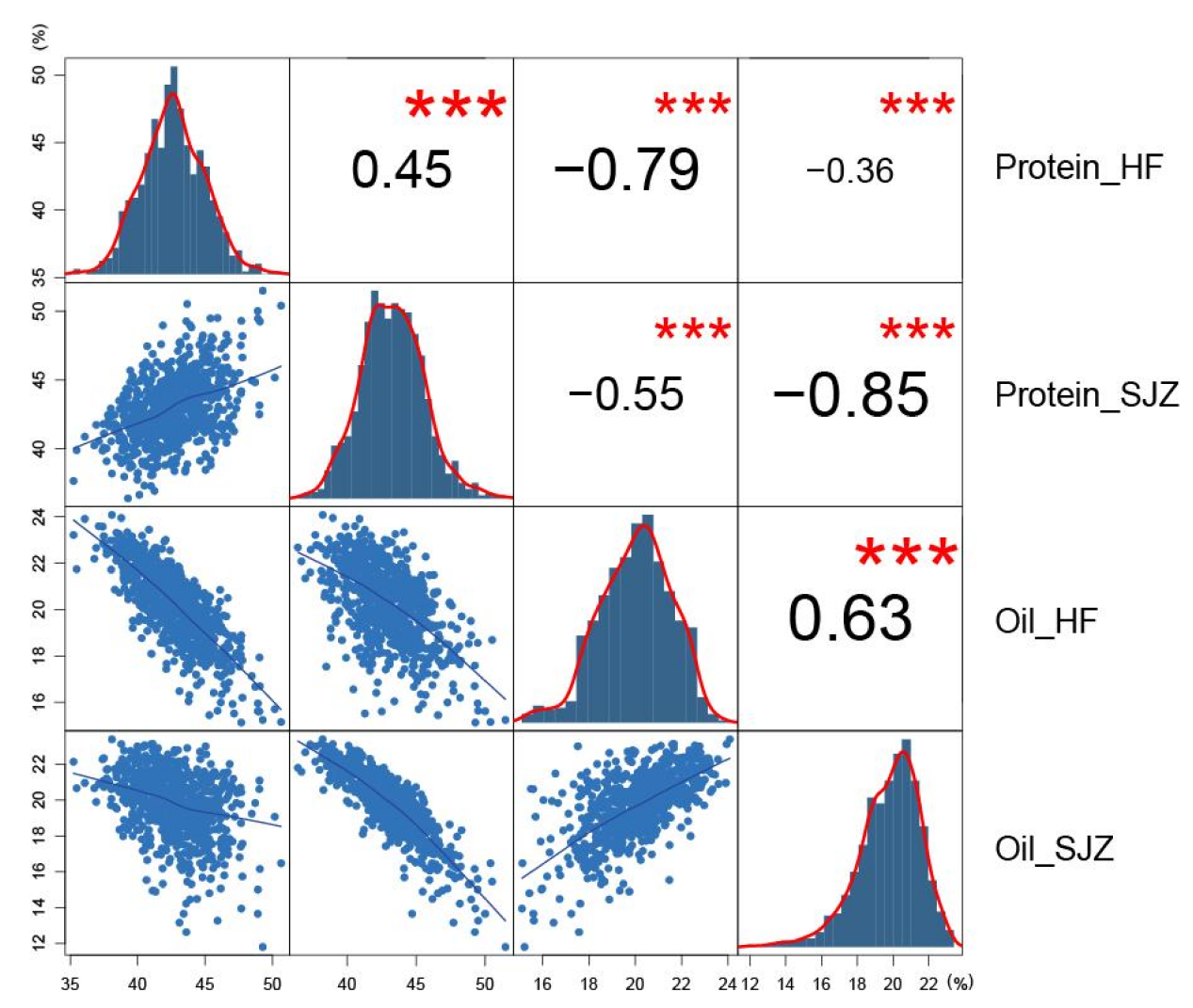


Figure S1. Correlation of oil and protein BLUP in Anhui and Hebei environments. Scatter plots on the lower left show the distribution between each pair of phenotypes. The diagonal displays the frequency distribution for each phenotype. Each blank space represents a phenotype, labeled on the right. The upper right contains the correlation coefficients (*R*) between each pair of phenotypes, with * indicating significant *P*-values, and *** indicating highly significant correlation.


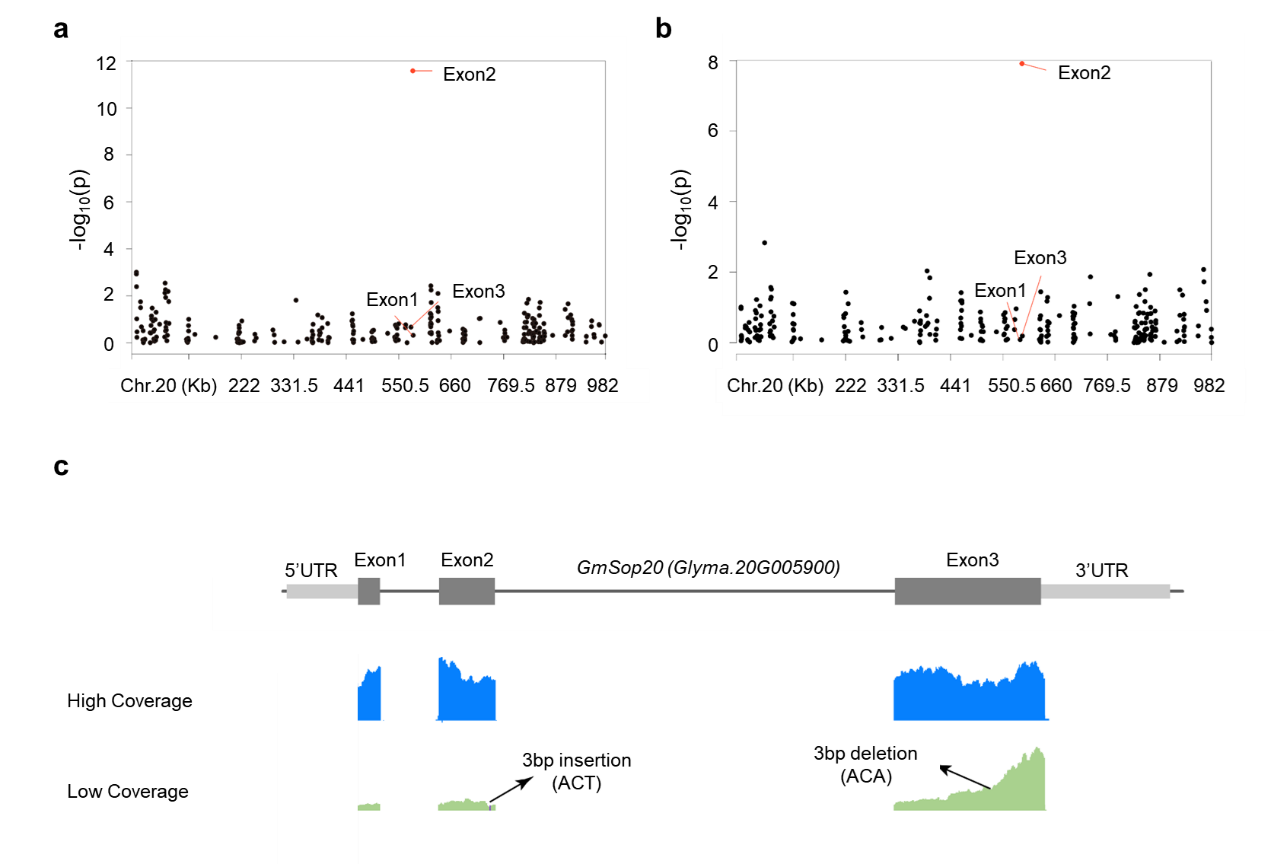


**Figure S2.** **Identification of *GmSop20* as a candidate gene regulating seed oil and protein content in soybean.** **a)** Seed oil content TWAS using a compressed mixed linear model. A 982 Kb window centered on *GmSop20* is shown. Each spot represents one gene or exon. **b)** Seed protein content TWAS using a compressed mixed linear model. A 982 Kb window centered on *GmSop20* is shown. Each spot represents one gene or exon. **c)** Two representative transcripts associated with oil and protein changes. Transcripts with normal expression levels are shown at the top (*GmSop20^Hap1^*), while abnormal transcripts are shown at the bottom (*GmSop20^Hap2/3^*). Five samples from each category were randomly selected and pooled for alignment visualization.


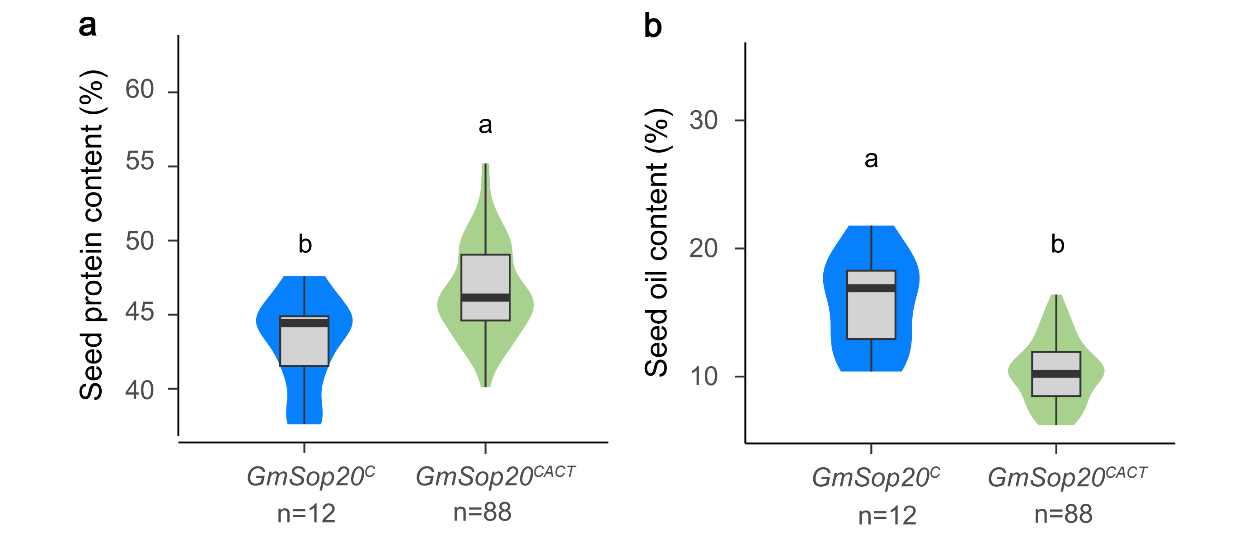


**Figure S3. Allelic effect of *GmSop20* on seed protein and oil content in wild soybean.** **a-b)** Distribution of seed traits for each haplotype, including seed protein content **(a)** and seed oil content **(b)**. The box plot shows the 25th to 75th percentile range, with a black line indicating the median. The whiskers extend to cover a range of 1.5 times the interquartile range, and black spots represent outliers. Statistical significance was determined by one-way ANOVA with Tukey’s multiple-comparison test.


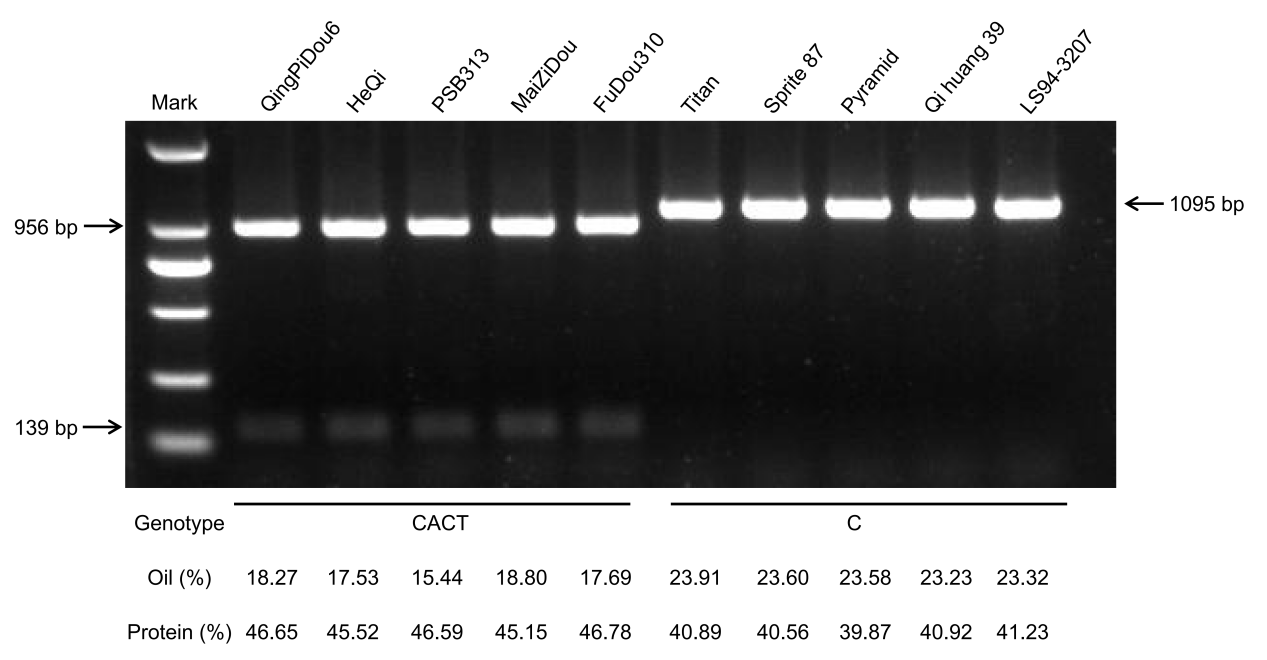


Figure S4. CAPS genotyping assay for ACT insertion in the coding region of *GmSop20*. PCR amplicons of 1095 bp were generated. The ACT insertion introduced an XspⅠ restriction site at this locus, resulting in digestion of the PCR products into two fragments of 956 bp and 139 bp. Genotypes of *GmSop20* and the corresponding seed oil and seed protein contents for each accession are shown. The data on seed oil and seed protein content are sourced from Table S2 (Supporting Information).


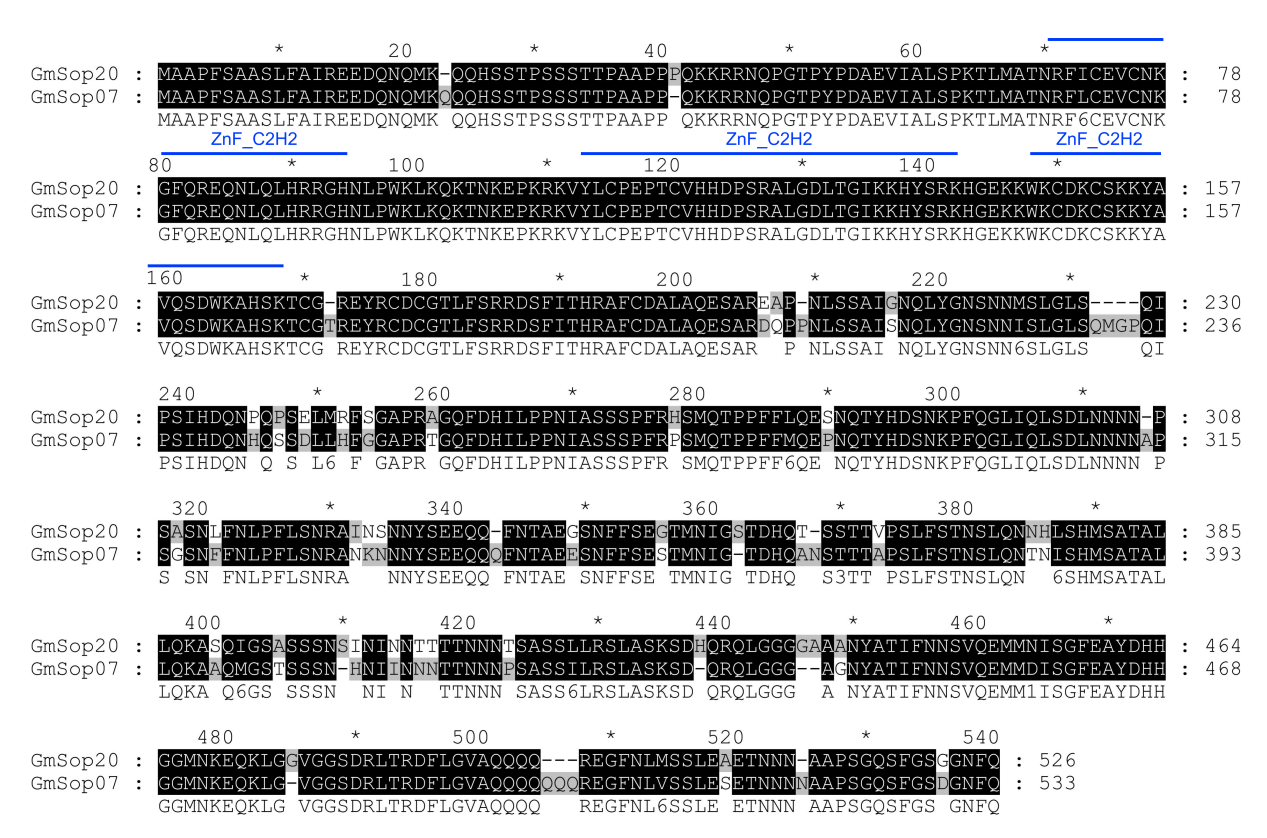


Figure S5. Alignment of amino-acid sequences of GmSop20 and GmSop07. The amino acid sequences were aligned by ClustalW Multiple alignments in MEGA7, and then manually adjusted using GeneDOC software. The sequences that encode the ZnF_C2H2 domain are marked in blue lines.


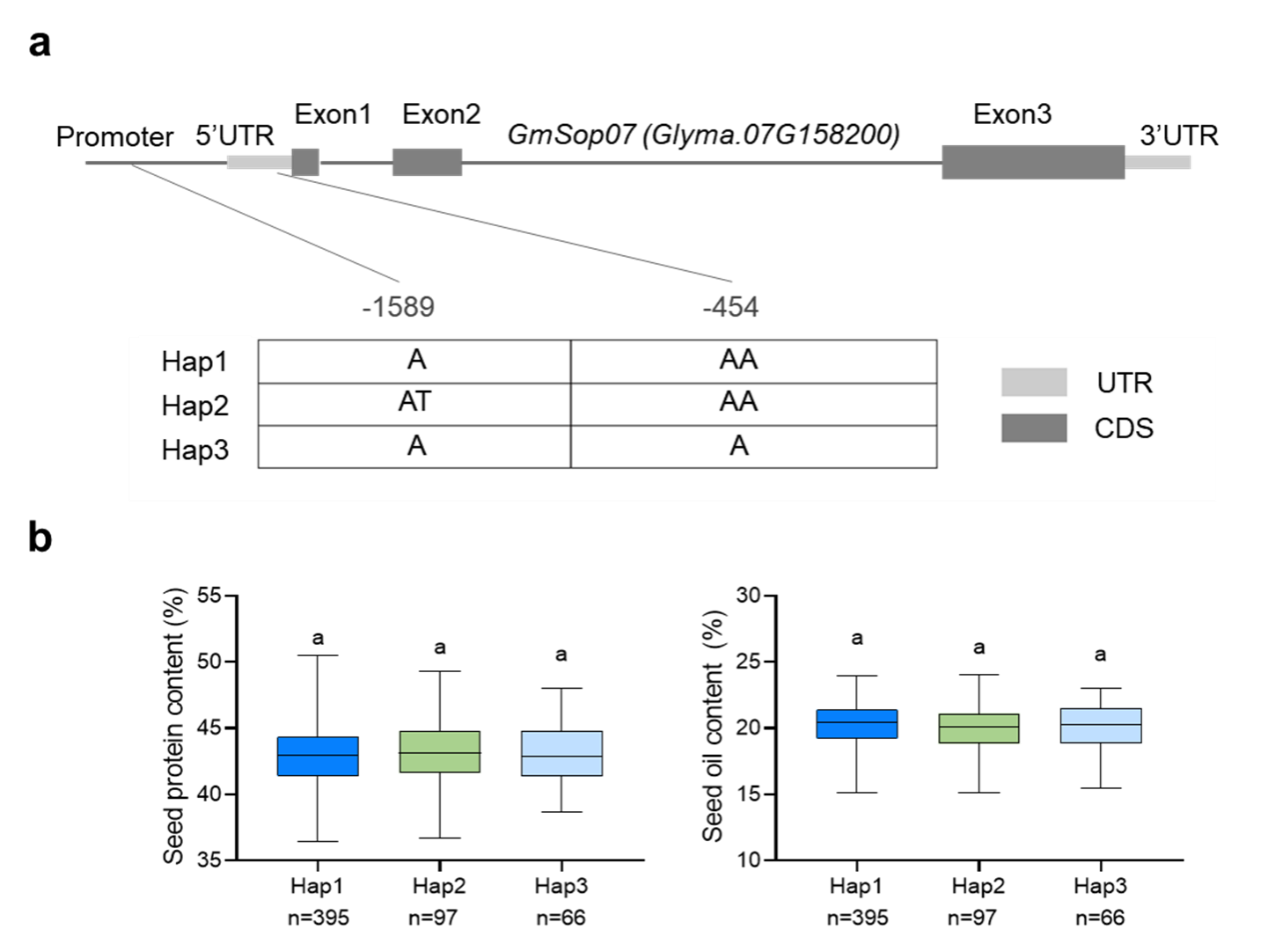


**Figure S6.** **Allelic effect of *GmSop07* on seed oil and protein content in cultivated soybean.** **a)** Haplotypes detected in the genomic region of *GmSop07*. **b)** Distribution of seed traits for each haplotype, including seed protein content (left) and seed oil content (right). The box plot shows the 25th to 75th percentile range, with the black line indicating the median. The whiskers extend to cover a range of 1.5 times the interquartile range, and black spots represent outliers. Statistical significance was determined by one-way ANOVA with Tukey’s multiple-comparison test.


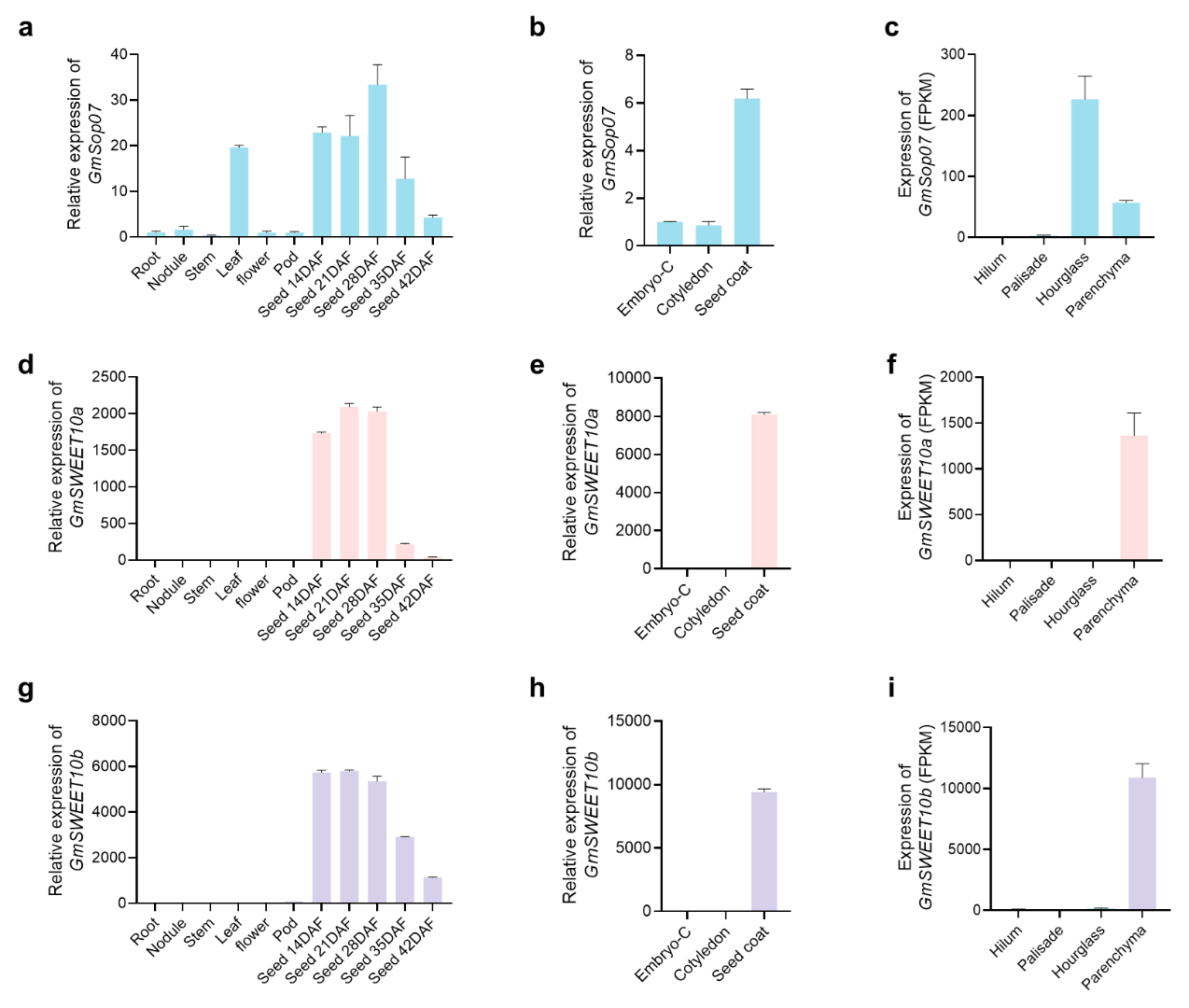


**Figure S7.** **The expression pattern of *GmSop07* and *GmSWEET10a/b*.** **a)** Transcript abundance of *GmSop07* in different organs. **b)** Transcript abundance of *GmSop07* in embryos with the cotyledon removed (Embryo-C), cotyledon, and seed coat. Developing seeds at 35 DAF were used for RNA extraction. **c)** Transcript abundance of *GmSop07* in Hilum, Palisade, Hourglass, and Parenchyma. **d)** Transcript abundance of *GmSWEET10a* in different organs. **e)** Transcript abundance of *GmSWEET10a* in embryos with the cotyledon removed (Embryo-C), cotyledon, and seed coat. Developing seeds at 35 DAF were used for RNA extraction. **f)** Transcript abundance of *GmSWEET10a* in Hilum, Palisade, Hourglass, and Parenchyma. **g)** Transcript abundance of *GmSWEET10b* in different organs. **h)** Transcript abundance of *GmSWEET10b* in embryos with the cotyledon removed (Embryo-C), cotyledon, and seed coat. Developing seeds at 35 DAF were used for RNA extraction. **i)** Transcript abundance of *GmSWEET10b* in Hilum, Palisade, Hourglass, and Parenchyma. (**c, f, i)** expression values were obtained from Gene Networks in Seed Development (<http://seedgenenetwork.net/soybean>). Data shown in (a-i) are mean ± SD (n=3).


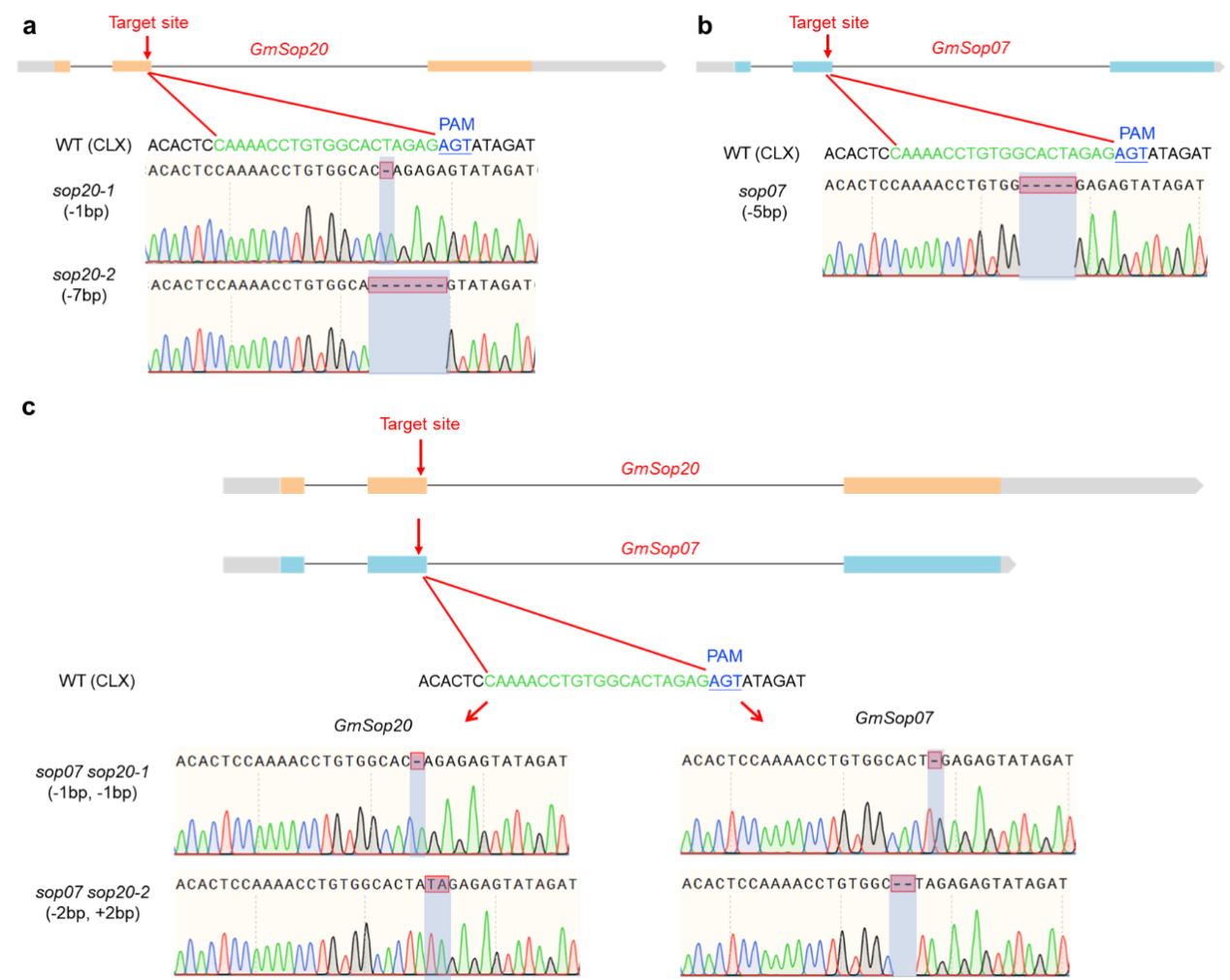


**Figure S8.** **Sanger sequencing for mutants created by genome editing.** **a)** Sanger sequencing for the mutants of *GmSop20*. WT refers to the sequence of wild-type CLX. *sop20-1* and *sop20-*2 refer to the two knockout mutants. **b)** Sanger sequencing for the mutants of *GmSop07*. WT refers to the sequence of wild-type CLX. *sop07* refers to the one knockout mutant. **c)** Sanger sequencing for the mutants of *GmSop20* and *GmSop07*. WT refers to the sequence of wild-type CLX. *sop07 sop20-1* and *sop07 sop20-*2 refer to the two double knockout mutants. CLX represents the JiLinCaiLiXiang soybean variety.


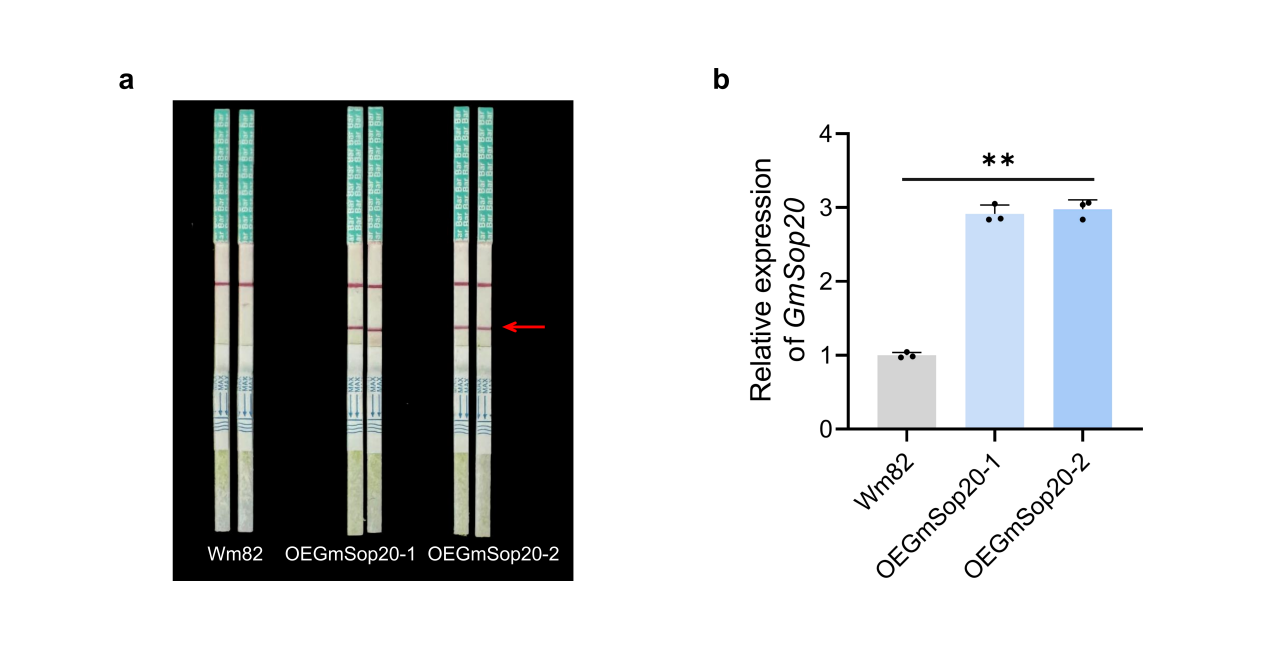


Figure S9. Identification of *GmSop20* overexpression lines. a) Identification of positive transgenic plants by strip detection for the presence of the selective bar gene. b) qPCR analysis of *GmSop20* in the overexpression lines. Data shown in (b) is mean ± SD (n=3). ** indicates *P*<0.01 of Student’s *t*-test between wild type (Wm82) and the overexpression lines. Wm82 represents the Williams 82 soybean variety.


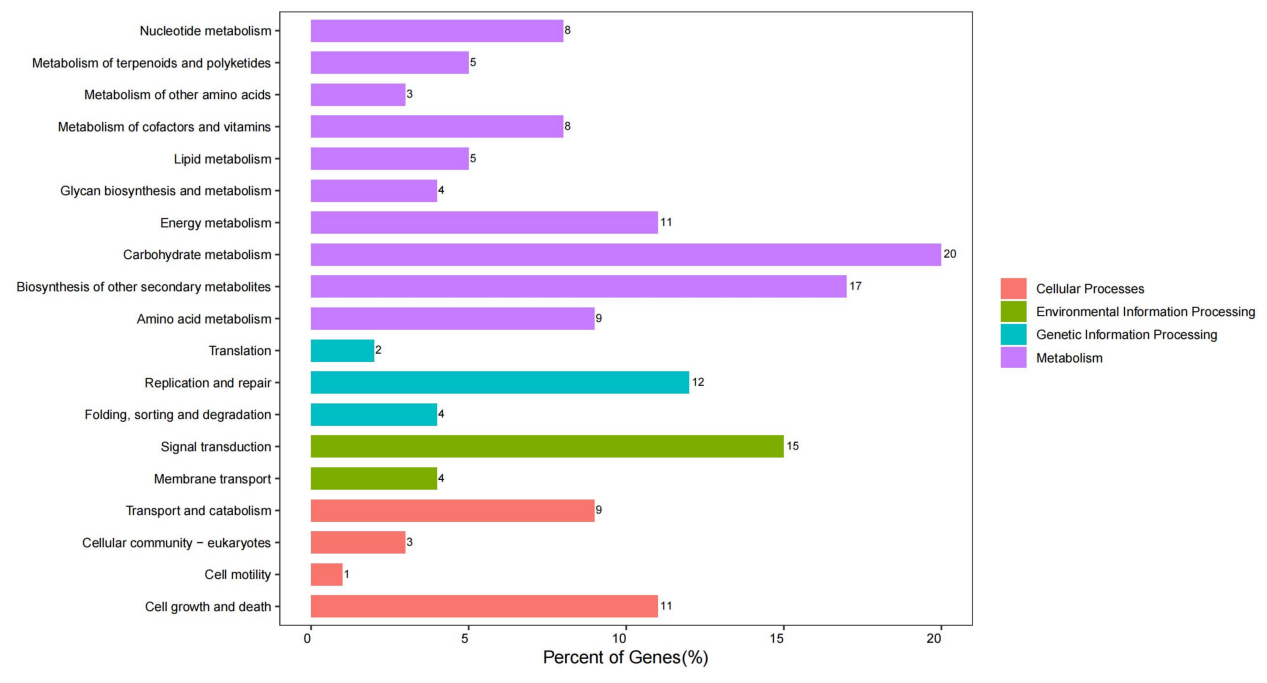


Figure S10. WT−vs−*sop20* (DOWN): KEGG Pathway Classification. Analysis was performed based on the Kyoto Encyclopedia of Genes and Genomes (KEGG) database (http://www.genome.jp/kegg/). The top 19 enriched KEGG pathways were ranked by *P*-values (*P*<0.05).


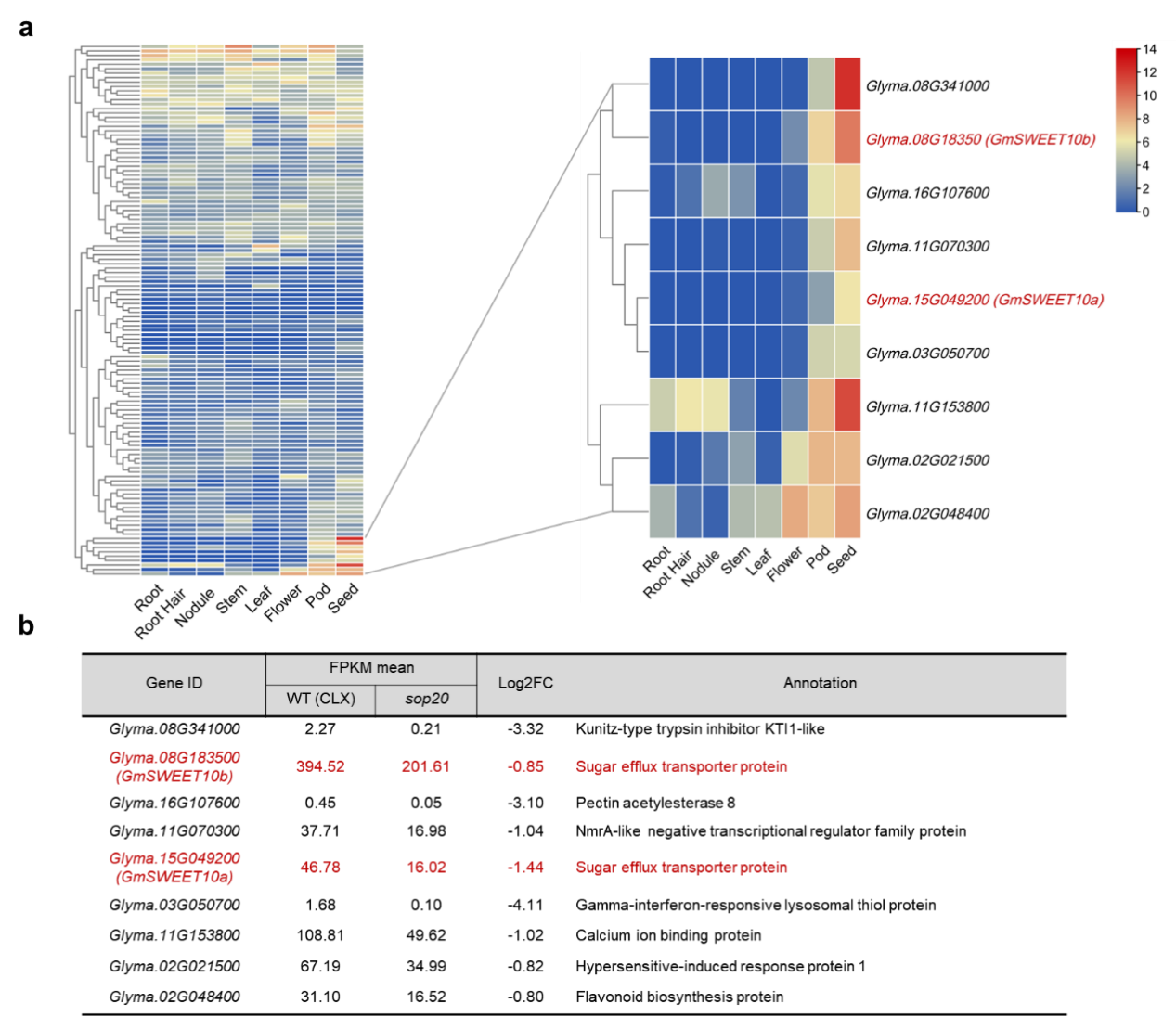


**Figure S11. Expression analysis of GmSop20 candidate target genes. a)** Heatmaps of candidate gene expression levels in different soybean tissues. The raw reads from RNA-seq data (https://phytozome-next.jgi.doe.gov/) were transformed to log2(FPKM+1). **b)** Functional categories of nine GmSop20 candidate target genes encoding for putative transporters. FPKM, fragments per kilobase of exon per million mapped. Fold change (FC) of the analyzed transcripts is based on RNA-seq data.


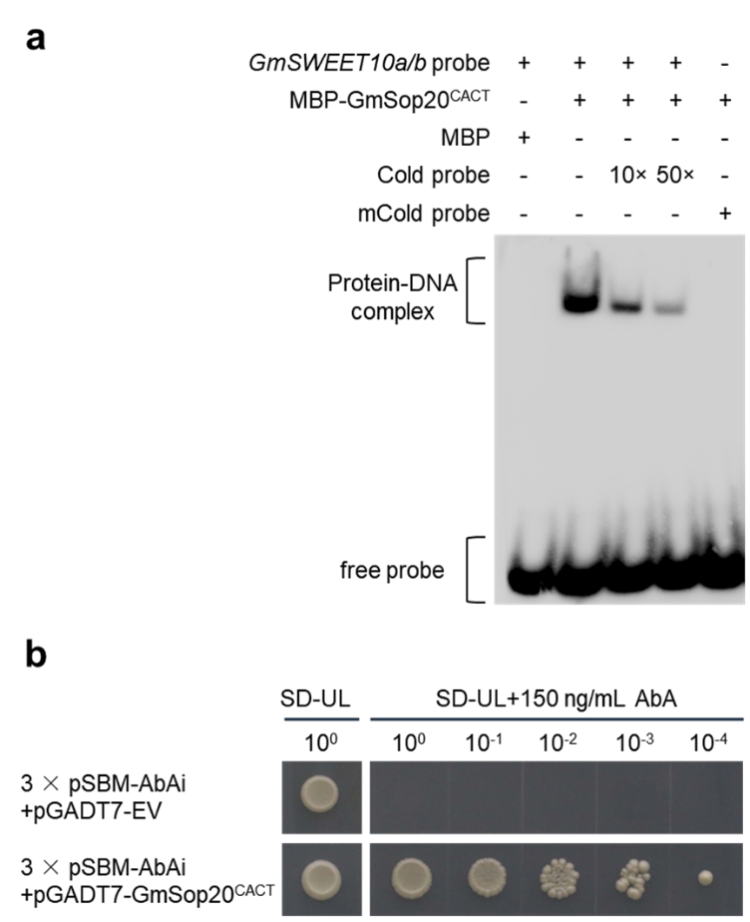


**Figure S12.** **GmSop20^CACT^ could bind to the *GmSWEET10a* and *GmSWEET10b* promoters.** **a)** An EMSA of MBP-GmSop20^CACT^ protein binding to the *GmSWEET10a/b* promoter. MBP or MBP-GmSop20^CACT^ fusion proteins were purified from *Escherichia coli*. Oligonucleotides (GCTCACACTATGA GACAAACGAGTA) were labeled with biotin. The probe sequence of *GmSWEET10a/b* was determined based on the location of the binding peaks in DAP-seq results and is listed in Table S11 (Supporting Information). An excess of non-labeled probe or mutated probe was used as controls. **b)** GmSop20^CACT^ can bind to the *GmSWEET10a/b* promoter sequence (CTATGAGACAAA) as confirmed by Y1H. (SBM: GmSop20 binding motif in *GmSWEET10a/b* promoter; SD-UL: SD/-Ura/-Leu).


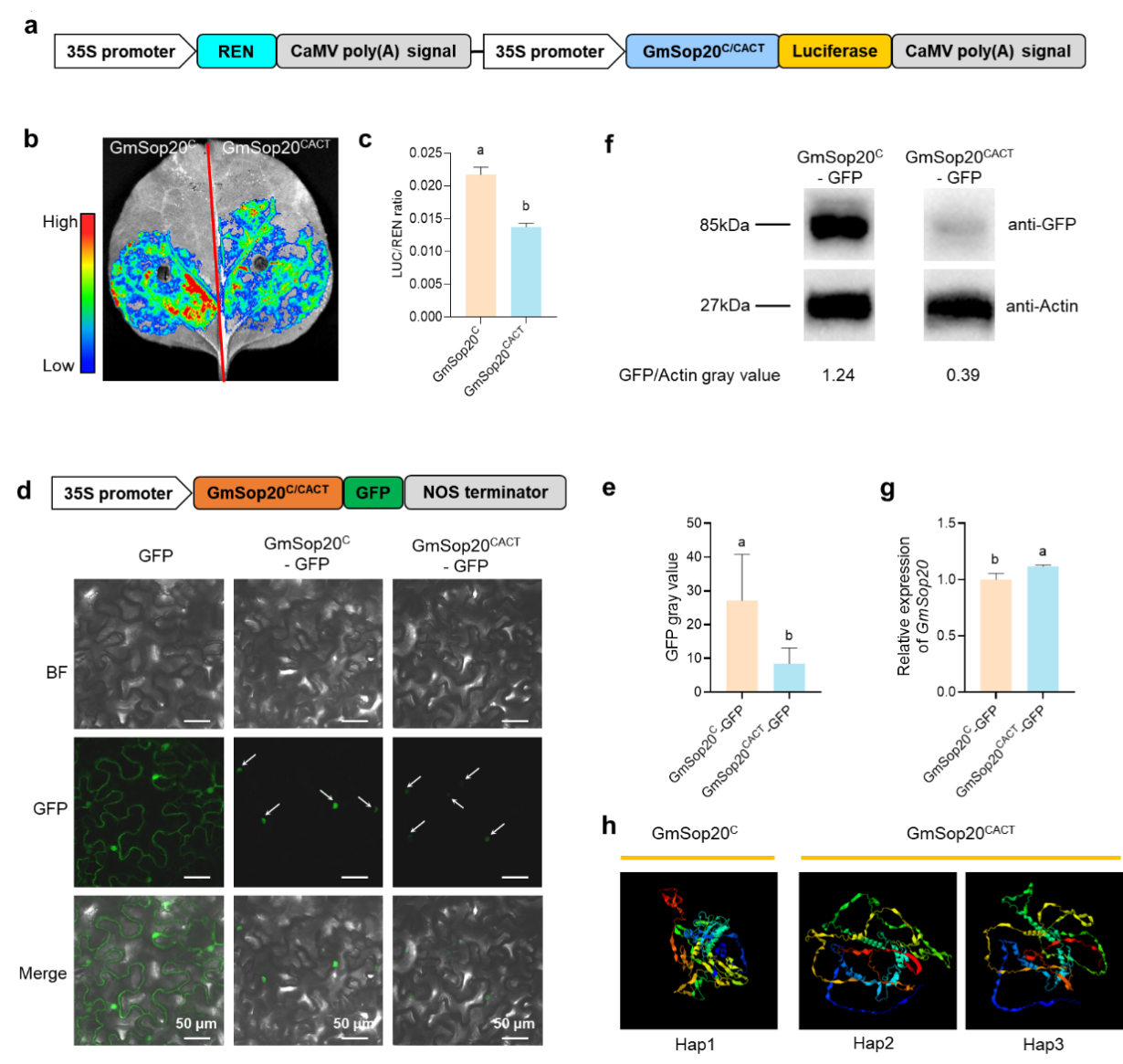


**Figure S13. GmSop20^C^ protein is more stable than the GmSop20^CACT^ protein.** **a)** Schematics of the constructs. **b)** The signal intensity shows the stability of different haplotypes of the GmSop20 protein. At least three independent experiments were performed. Similar results were observed. **c)** Quantification of the signal intensity based on LUC/REN ratios. **d)** The green signal in the GFP channel shows fluorescence from 35S-GFP or GmSop20^C/CACT^-GFP fusion protein. Scale bars, 50 μm. Abbreviations: BF, bright field; GFP, green fluorescent protein. Merge, merged images of GFP and BF channels are shown. Experiments were repeated three times. **e)** The green fluorescent value of the fusion protein from the two GmSop20 haplotypes was determined using Image J software. **f)** The abundance of the protein product from the two GmSop20 haplotypes was determined using Image J software. **g)** Analysis of transcript abundance of GmSop20^C^/GmSop20^CACT^ in *N. benthamiana* leaves. *N. benthamiana* leaves were collected 48 hours post-injection for RNA extraction. **h)** Predicted protein structural difference between GmSop20^C^ (GmSop20^Hap1^) and GmSop20^CACT^ (GmSop20^Hap2/3^) by the D-I-TASSER server. Data shown in (c, e, g) are mean ± SD (n≥3). Statistical significance was determined by one-way ANOVA with Tukey’s multiple-comparison test.


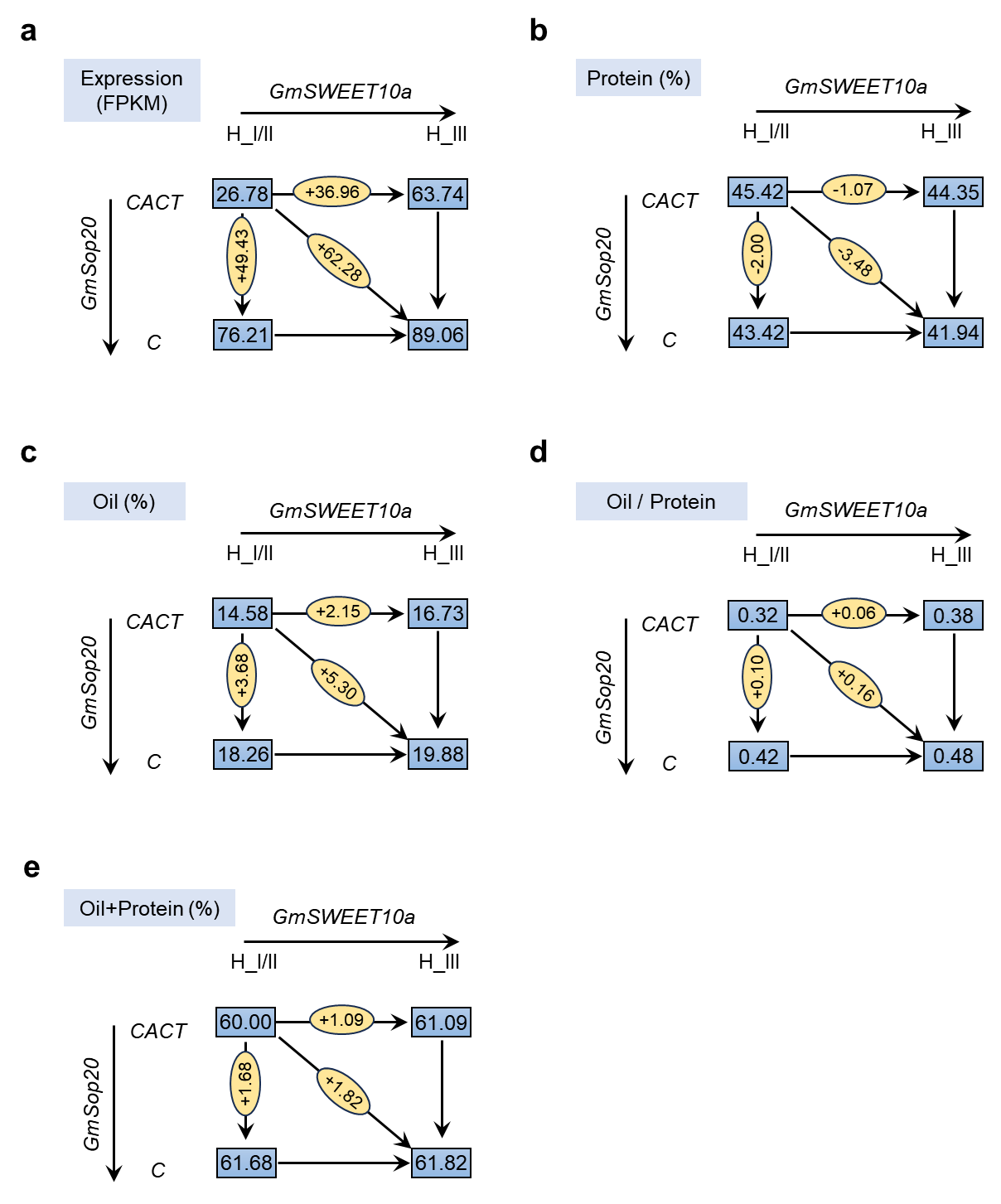


Figure S14. Correlation analysis of the *GmSop20* and *GmSWEET10a* alleles with the related phenotypes. a-e) By comparing the phenotypes before and after artificially selections, the mean value and their difference were calculated with the expression level of *GmSWEET10a* (a) protein content (b), oil content (c), the ratio of oil and protein (d), and the sum of oil and protein content (e).


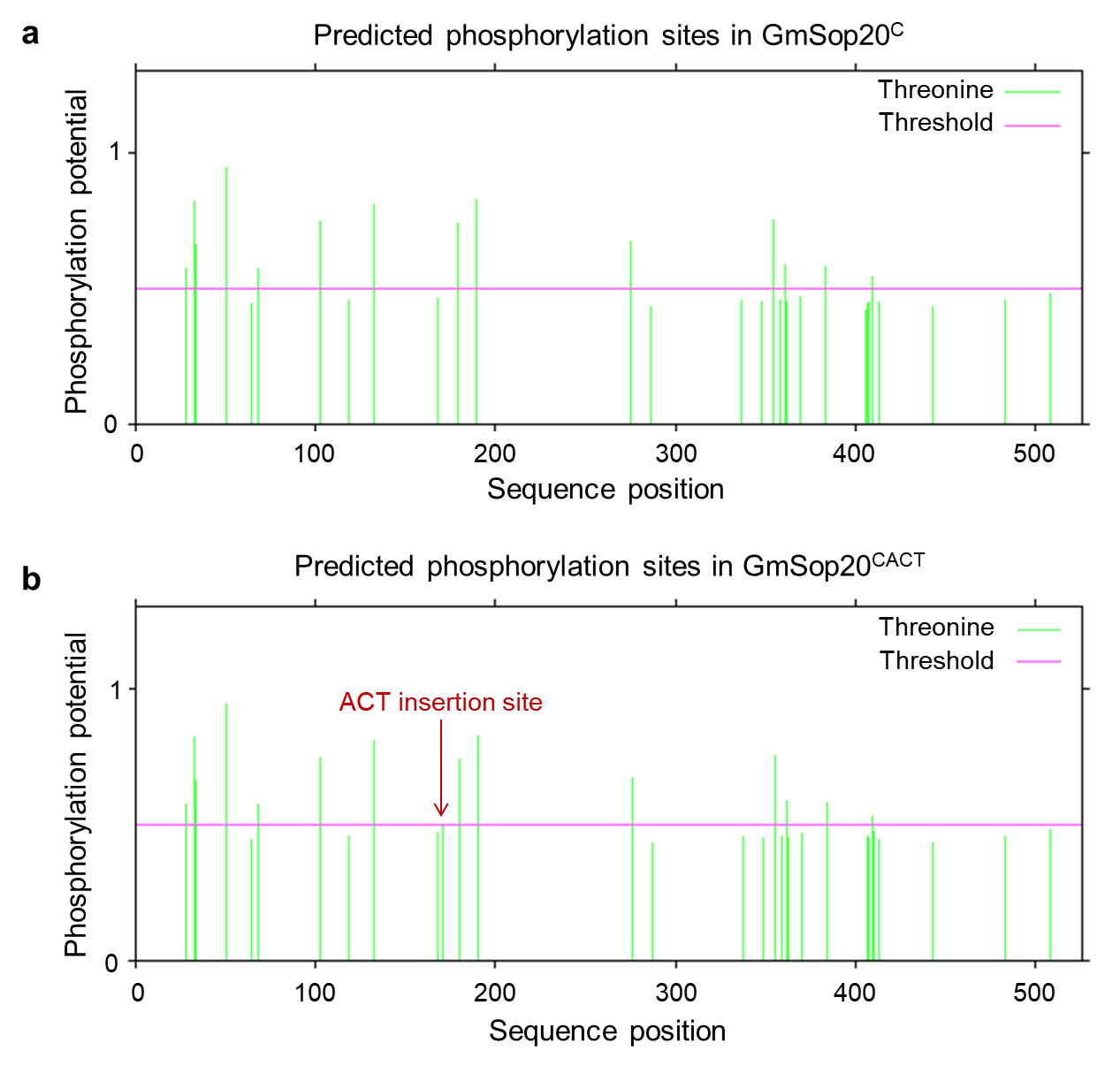


Figure S15. Phosphorylation site prediction of GmSop20^C^ and GmSop20^CACT^. a-b) Predicted differences in protein phosphorylation sites between GmSop20^C^ (a) and GmSop20^CACT^ (b), as determined by NetPhos 3.1.


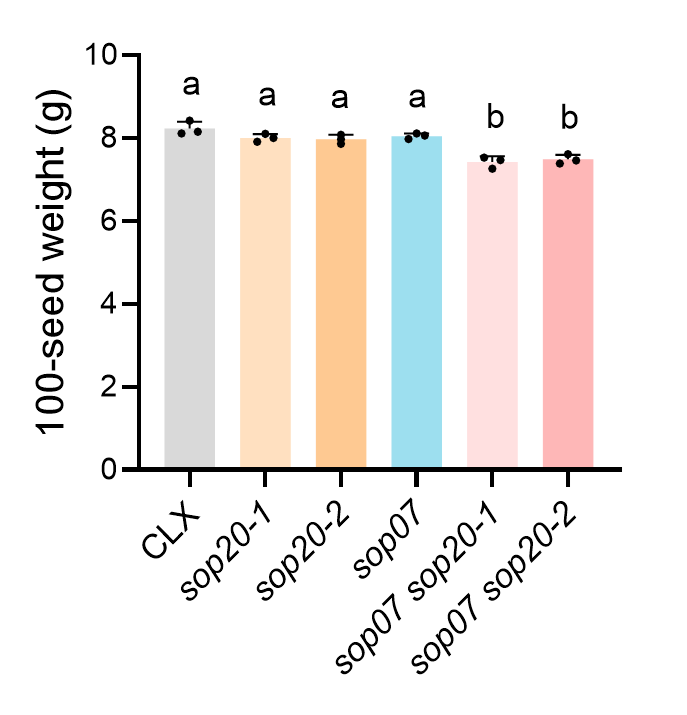


**Figure S16. Effect of *GmSop20* and *GmSop07* on seed weight.** 100-seed weight of mature seeds from wild type (CLX), *sop20*, *sop07*, double mutant *sop07 sop20*. Data shown in figure represent mean ± SD (n=3). Statistical significance was determined by one-way ANOVA with Tukey’s multiple-comparison test.

Supporting Information

Figure S1. Correlation of oil and protein BLUP in Anhui and Hebei environments.

Figure S2. Identification of *GmSop20* as a candidate gene regulating seed oil and protein content in soybean.

Figure S3. Allelic effect of *GmSop20* on seed oil and protein content in wild soybean.

Figure S4. CAPS genotyping assay for ACT insertion in the coding region of *GmSop20*.

Figure S5. Alignment of amino-acid sequences of GmSop20 and GmSop07.

Figure S6. Allelic effect of *GmSop07* on seed oil and protein content in cultivated soybean.

Figure S7. The expression pattern of *GmSop07* and *GmSWEET10a/b*.

Figure S8. Sanger sequencing for mutants created by gene editing.

Figure S9. Identification of *GmSop20* overexpression lines.

Figure S10. WT−vs−*sop20* (DOWN): KEGG Pathway Classification.

Figure S11. Expression analysis of GmSop20 candidate target genes.

Figure S12. GmSop20^CACT^ could bind to the *GmSWEET10a* and *GmSWEET10b* promoters.

Figure S13. GmSop20^C^ protein is more stable than the GmSop20^CACT^ protein.

Figure S14. Correlation analysis of the *GmSop20* and *GmSWEET10a* alleles with the related phenotypes.

Figure S15. Phosphorylation site prediction of GmSop20^C^ and GmSop20^CACT^.

Figure S16. Effect of *GmSop20* and *GmSop07* on seed weight.

Table S1. Accessions used for GWAS and TWAS in this study.

Table S2. Haplotype, phenotype, geographic, and expression information of the soybean accessions in this study.

Table S3. Heritability of oil and protein.

Table S4. List of oil and protein loci identified by GWAS.

Table S5. 365 soybean accessions with RNA-Seq data.

Table S6. List of oil and protein loci identified by TWAS.

Table S7. Amino acid sequence of mutants.

Table S8. RNA-seq profiling of differentially expressed genes in *sop20* mutant seeds.

Table S9. Genes associated with GmSop20 binding sites identified by DAP-seq.

Table S10. RNA-seq and DAP-seq co-identified the GmSop20 target genes.

Table S11. Primers and probes used in this study.
